# A Metatranscriptomics Survey of Microbial Diversity on Surfaces Post-Intervention of cleanSURFACES® Technology in an Intensive Care Unit

**DOI:** 10.3389/fcimb.2021.705593

**Published:** 2021-07-20

**Authors:** Jeremy Chen See, Truc Ly, Alexander Shope, Jess Bess, Art Wall, Saketram Komanduri, John Goldman, Samantha Anderson, Christopher J. McLimans, Colin J. Brislawn, Vasily Tokarev, Justin R. Wright, Regina Lamendella

**Affiliations:** ^1^ Contamination Source Identification, Huntingdon, PA, United States; ^2^ AIONX, Hershey, PA, United States; ^3^ Nextflex, San Jose, CA, United States; ^4^ UPMC Harrisburg, Harrisburg, PA, United States; ^5^ Department of Biology, Juniata College, Huntingdon, PA, United States

**Keywords:** continuous cleaning, hospital, HAI, metatranscriptomics, NGS

## Abstract

Hospital-acquired infections (HAIs) pose a serious threat to patients, and hospitals spend billions of dollars each year to reduce and treat these infections. Many HAIs are due to contamination from workers’ hands and contact with high-touch surfaces. Therefore, we set out to test the efficacy of a new preventative technology, AIONX^®^ Antimicrobial Technologies, Inc’s cleanSURFACES^®^, which is designed to complement daily chemical cleaning events by continuously preventing re-colonization of surfaces. To that end, we swabbed surfaces before (Baseline) and after (Post) application of the cleanSURFACES^®^ at various time points (Day 1, Day 7, Day 14, and Day 28). To circumvent limitations associated with culture-based and 16S rRNA gene amplicon sequencing methodologies, these surface swabs were processed using metatranscriptomic (RNA) analysis to allow for comprehensive taxonomic resolution and the detection of active microorganisms. Overall, there was a significant (P < 0.05) global reduction of microbial diversity in Post-intervention samples. Additionally, Post sample microbial communities clustered together much more closely than Baseline samples based on pairwise distances calculated with the weighted Jaccard distance metric, suggesting a defined shift after product application. This shift was characterized by a general depletion of several microbes among Post samples, with multiple phyla also being reduced over the duration of the study. Notably, specific clinically relevant microbes, including *Staphylococcus aureus*, *Clostridioides difficile* and *Streptococcus* spp., were depleted Post-intervention. Taken together, these findings suggest that chemical cleaning events used jointly with cleanSURFACES^®^ have the potential to reduce colonization of surfaces by a wide variety of microbes, including many clinically relevant pathogens.

## Introduction

A strong link between contamination of environmental surfaces in healthcare settings, especially surfaces that are most often touched (i.e., over-the-bed-tables, keyboards, etc.), and incidence of hospital acquired infections (HAIs) is well-documented (Rheinbaben et al., 2000; Weber et al., 2010; CDC, 2015; Mitchell et al., 2015; Anderson et al., 2018). Studies published by the Centers for Disease Control and Prevention (CDC) have found that hospitals spend more than $10 billion annually on direct medical expenses to treat HAIs (Scott II, 2009). Additionally, 20% to 40% of HAIs arise *via* the contaminated hands of workers and high-touch environmental surfaces (Weber et al., 2010). Surfaces are typically cleaned manually with liquid chemical disinfectants once per day; however, this acts only in the moment and provides limited lasting effects. The moment another patient, employee, or visitor enters the cleansed space the surfaces will be re-contaminated and will remain contaminated until the next cleaning (Kramer and Assadian, 2014). The Hospital Microbiome Project (Ramos et al., 2015) revealed that samples from surfaces, air, and water reveal a quickly changing microbiome due to the constant shedding of microbes from individuals populating that environment. With the established link between environmental contamination and HAIs, these episodic cleaning mechanisms fall far short of filling the apparent need.

Current disinfection literature has largely addressed the impact of various cleaning methods (disinfection sprays, wipes, ultraviolet treatment) through the cultivation of microbes (Stewart et al., 2014; Kwan et al., 2018). While these efficacy results are promising, several studies have highlighted concerns surrounding inadequate cleaning and subsequent recontamination (Stewart et al., 2014; Kwan et al., 2018). Considering the antimicrobial properties inherent to copper, studies have shown copper surfaces to be a promising alternative in maintaining a persistent disinfecting surface (Schmidt et al., 2012; Bogdanović et al., 2014). AIONX^®^ Antimicrobial Technologies, Inc. has developed a unique technology, cleanSURFACES^®^, which harness the inherent antimicrobial properties of silver and copper optimizing them *via* a micro-electric current to their ionic states. This forced ionization releases the proper concentrations of the metal ions, creating a toxic environment for pathogenic organisms while remaining low enough to remain harmless to human cells. The proprietary design of cleanSURFACES^®^ creates an open circuit with a silver/copper embedded paste. This open circuit closes when pathogens, respiratory droplets, or other objects contact the surface, allowing the electrical current to flow. Herein, we evaluated the efficacy of cleanSURFACES^®^, a continuous-cleaning product by AIONX^®^ for use on high-touch surfaces in an Intensive Care Unit in UPMC Harrisburg (Harrisburg, PA).

Sequencing the microbial community of surface swabs is one way to evaluate the cleanliness of surfaces. Next-generation sequencing (NGS) technologies have enabled up to billions of DNA fragments to be sequenced in a high-throughput, multiplexed, and parallelized manner. Recently NGS technologies, such as shotgun metagenomics (MG) and metatranscriptomics (MT), have been applied to environmental microbiological testing, which have enabled a comprehensive and less biased approach to pathogen detection (Ramos et al., 2015; Tang et al., 2015; Batovska et al., 2019; Comar et al., 2019; Rampelotto et al., 2019; Mohan et al., 2020). Comprehensive testing methods like MG and MT randomly sequence DNA and RNA, respectively, from a sample and cast an untargeted net for capturing microbial (prokaryotic and eukaryotic) diversity in complex environments. A recent study compared hospital bacterial community compositions based on culturing, qPCR, and 16S rRNA gene amplicon sequencing, with 16S rRNA gene amplicon sequencing proving to be the more sensitive and comprehensive as compared to other targeted PCR-based methods (Comar et al., 2019). However, it should be noted that 16S rRNA gene amplicon sequencing has several important limitations, including an inability to differentiate between active/inactive cells or viruses, as well as low taxonomic resolution. More recently, a similar study compared 16S rRNA gene amplicon sequencing and shotgun metagenomics, and over twice as many microbial genera were able to be identified through metagenomics (Mohan et al., 2020). Therefore, untargeted methods, such as MG and MT could prove even more valuable in contamination detection in a hospital setting. While MG is unable to differentiate between active and potentially latent/dead microbial populations and metabolic pathways from a given sample, MT analyses can provide insight to active genes and microbes. In a clinically relevant setting, MT analysis presents a unique opportunity in the identification of active pathogenic organisms and antimicrobial-resistance mechanisms (Wilson et al., 2018; Huang et al., 2019).

Accordingly, we applied a novel metatranscriptomics sequencing workflow (CSI-Dx™) to characterize active microbial community composition through the taxonomic annotation of all expressed genes. The active microbial communities were then analyzed to evaluate AIONX^®^’s continuous-cleaning technology’s effectiveness for reducing environmental surface contamination over time in an intensive care unit (ICU).

## Methods

### Site Information

**Figure 1:**
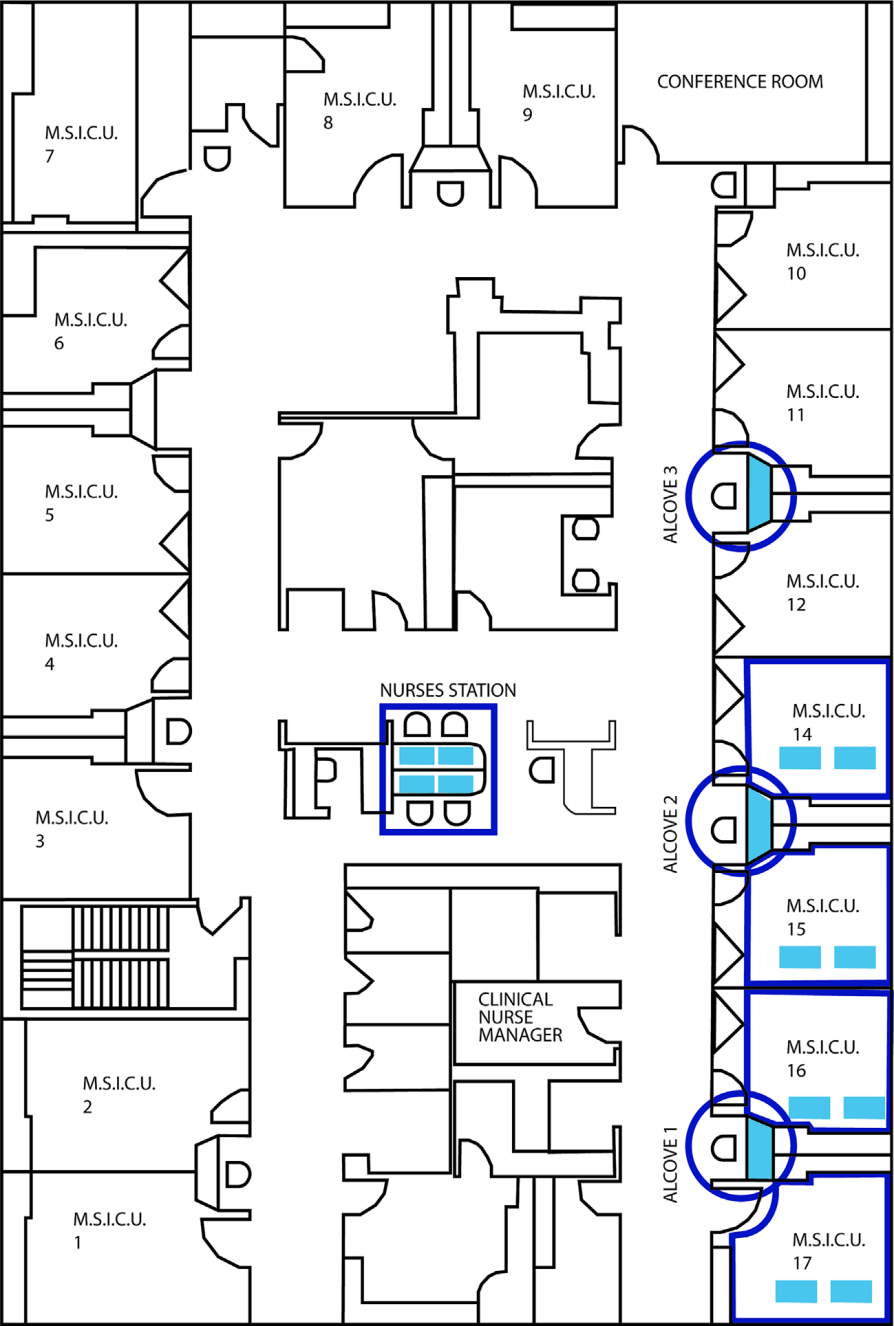
Detailed blueprint of the ICU layout illustrating the location of the 4 patient rooms, 3 nurse’s alcoves (circles) and central nurses station samples during the study.

This study was performed at UPMC Harrisburg (Harrisburg, PA) in their medical intensive care unit (ICU). While there were other ICUs (surgical and neonatal) at UPMC Harrisburg, this medical ICU was selected for the following two reasons: high average census, and most acutely ill patients were sent to this medical ICU. Pre-intervention (Baseline) samples established the microbial community of the studied areas after routine cleaning and before placement of the cleanSURFACES^®^. After review of the ICU, 22 high-touch surfaces were identified for inclusion in the study. These surfaces were in four specified patient rooms, three nurse alcove workstations between patient rooms, and four central staff/nurse workstations (see **Figure 1** for detailed ICU layout). A total of 88 Baseline samples were collected from 22 surfaces, with each sampled at 0, 2, 4, and 8 hours following a routine hospital cleaning event (for sampled surfaces see **Table 1**).

**Table 1:** Summary of collection sites, surfaces sampled, and sampling time points during the study.

Collection Site	Number of Sites Assessed	Covered Surfaces Sampled Per Site	Uncovered Surfaces Sampled Per Site	Sampling Time points	Total Surfaces Sampled Per Time point
*Patient Room*	4	Over Bed Table [OBT] (N = 1)	WOW Keyboard (N = 1)	Baseline, Day 1, Day 7, Day 14, Day 28	8
*Nurse’s Alcove*	3	Worksurface (N = 1)	Keyboard (N = 1)	Baseline, Day 1, Day 7, Day 14, Day 28	6
*Central Nurse Station*	4	Worksurface (N = 1)	Keyboard (N = 1)	Baseline, Day 1, Day 7, Day 14, Day 28	8
					Total Number of Unique Sampled Surfaces = 22

**Figure 2:**
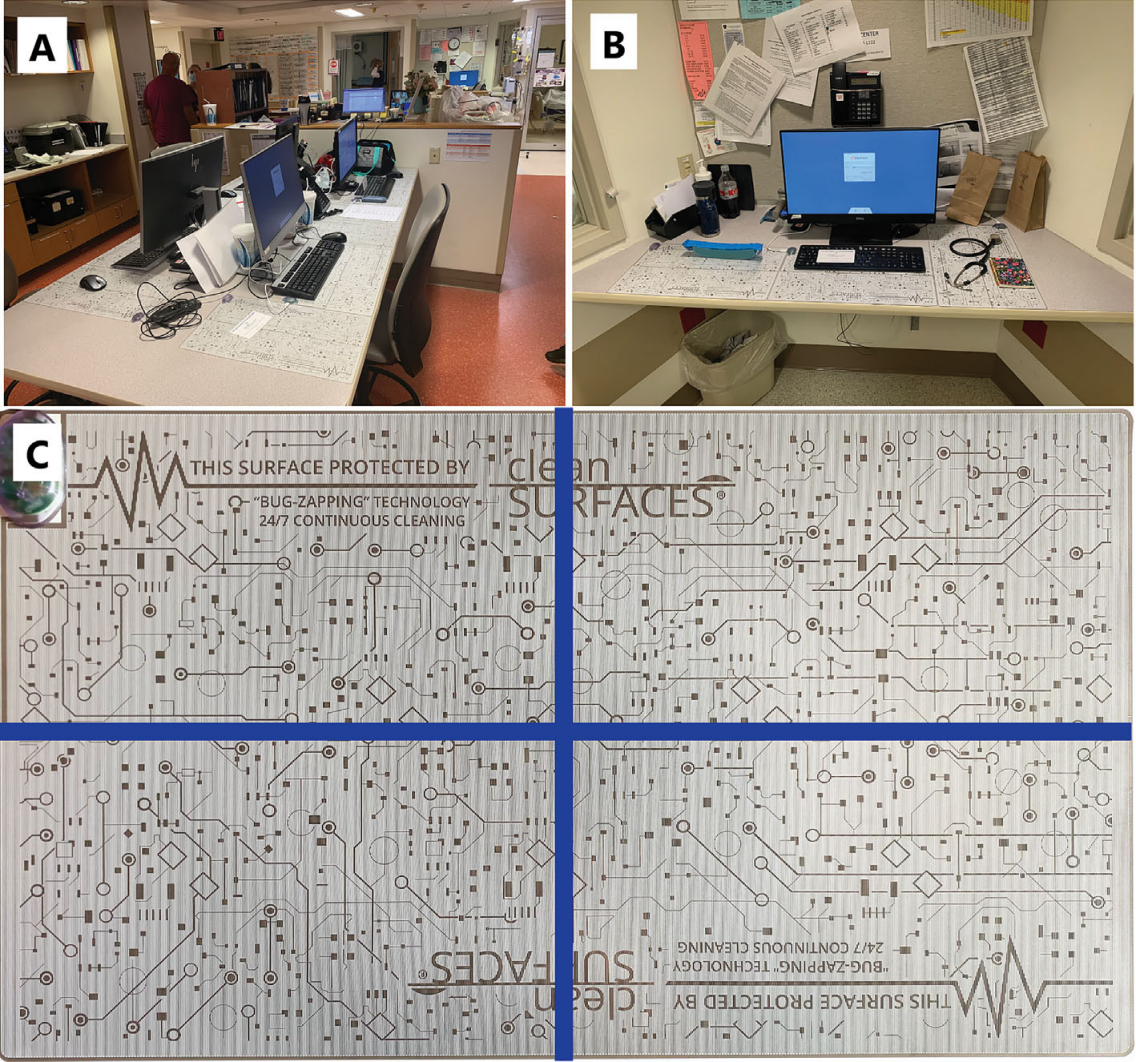
Pictures depicting the placement of cleanSURFACES^®^ technology around the ICU. **(A)** Central nurse’s workstation for the ICU with cleanSURFACES^®^ in place on the work area of each of the 4 stations, **(B)** placement of cleanSURFACES^®^ in alcove workstations between test rooms, **(C)** division of a cleanSURFACES^®^ product into 4 quadrants for sampling on different days in the post-installation period of the study.

cleanSURFACES^®^ mats were installed on several work surfaces in the central staff/nurse work area, in addition to each nurse alcove between patient rooms. Mats were also placed on surfaces in four patient rooms including: Over-the-bed-table (OBT) and flat areas atop computer stations or workstation on wheels (WOW) (see **Figures 2A, B**). cleanSURFACES^®^ were installed and covered flat surfaces like OBT and workstations (WS). Conversely, other surfaces like keyboards and WOW keyboards were unable to be covered and cleanSURFACES^®^ mats were subject to placement either below or at an accessible location near the keyboard. With cleanSURFACES^®^ mats designed to function for at least 60 days, all 22 surfaces that were swabbed for Baseline samples were also swabbed post-installation at 1, 7, 14, and 28 days. **Table 1** includes details on surfaces that were either covered or uncovered with the cleanSURFACES^®^ product and swabbed. Once samples were collected, swabs were immediately transported to the Contamination Source Identification (CSI) laboratory for processing and analysis.

Cleaning protocols varied for surfaces inside patient rooms and for those outside the rooms. During patient stays, high touch surfaces in patient rooms were cleaned once a day by environmental services (EVS) using a standard quaternary ammonium compound chemical disinfectant. Rooms with COVID-19 patients, however, did not receive this daily cleaning. Upon patient discharge, rooms received a more thorough terminal cleaning by EVS. All high touch surfaces were cleaned, including all surfaces involved in the present study. Surfaces outside patient rooms were cleaned by staff (not including EVS) regularly. Staff members used pre-moistened chemical cleaners (quaternary ammonium compounds, bleach, or hydrogen peroxide) to periodically wipe work areas. Frequency of cleaning varied among staff members but should have remained consistent throughout the study.

### Sample Collection

Swabbing events during the study were conducted in a standardized manner adapted from the methodology described in the CDC guidelines (National Institute for Occupational Safety and Health, 2010). Samples were collected using CaptiSwabs (Captigen, LLC, Philadelphia, PA). After sampling of the respective surface, swabs were ejected into 50 mL conicals containing 2 mL of DNA/RNA Shield (Zymo Research, Irvine CA) and were stored at ambient temperatures up to 30 days as suggested by Zymo Research, until RNA extraction was performed.

A total of 88 Baseline and 88 Post-intervention samples were collected from 22 surfaces. Each of the 22 surfaces were divided into quadrants and swabbed to generate Baseline samples after routine cleaning in the following order: Hour 0 (immediately following cleaning event) - Quadrant 1, Hour 2 - Quadrant 2, Hour 4 - Quadrant 3, Hour 8 - Quadrant 4. Once cleanSURFACES^®^ mats were installed, they were also divided quadrants (see **Figure 2C**) and swabbed for a total of 88 Post-intervention samples in the following order: Day 0- Quadrant 1, Day 7- Quadrant 2, Day 14- Quadrant 3, Day 28- Quadrant 4. All Post-intervention samples were taken 6 hours post-cleaning, since prior studies on this subject suggest that contamination levels would return to pre-cleaning levels by 6 hours on average (Wilson et al., 2011; Russotto et al., 2015). Different quadrants of cleanSURFACES^®^ mats were sampled for each time point since evidence suggests that swabs remove portions of bioburden from surfaces and can ultimately introduce sampling bias (Moore and Griffith, 2007; Landers et al., 2010). Baseline samples were the only controls (swabs from surfaces only treated with chemical disinfectant) collected during the study; no additional control samples were collected in parallel with Post-intervention samples.

During each swabbing event, trained swabbing personnel began with the upper corner of the quadrant being sampled and proceeded in a serpentine pattern diagonally to the opposite corner using a flat side of the CaptiSwab. The swab was then flipped, and the pattern was repeated beginning in the contralateral corner and proceeding diagonally in the same winding pattern until the opposing corner was reached. When sampling keyboards, each key in the quadrant being sampled was swabbed in a clockwise manner utilizing the tip of the swab to interact with the concave key surfaces. Three consecutive clockwise circles were performed before moving onto the next key in the quadrant. All corners of the swab were utilized to maximize specimen capture. Care was taken to not sample in between the keys and to only obtain samples from the key surfaces.

### RNA Extraction, Concentration, and Quantification

RNA from each swab sample was isolated by extracting 1 mL of DNA/RNA Shield with a Zymobiomics DNA/RNA Miniprep kit (Zymo Research, Irvine CA). All samples from a time point were extracted in a single set and independently from other time points with the addition of a single no template control (NTC) to control for any introduced contamination. Using the manufacturer’s instructions, RNA was concentrated using the Zymobiomics RNA Clean & Concentrator-5 kit (Zymo Research, Irvine CA). Concentrated RNA was quantified using Quant-iT 1X dsDNA High Sensitivity Assay (Thermo Fisher Scientific, Waltham MA) to confirm DNase treatment and a Quant-iT RNA Assay was used to measure RNA concentrations (**Supplemental Table 1**). Based on the DNA concentrations being below detection for all samples, it was assumed that the data generated after cDNA sequencing was from cDNA, not residual DNA. However, there is no current bioinformatic methodology by which sequence reads can be categorized as originating from DNA or RNA genomic content. Both assays were performed on a Tecan Infinite 200 PRO plate reader (Tecan US, Morrisville NC). Any extracts that yielded detectable RNA were normalized to 500 pg (0.5 ng) input for each downstream cDNA synthesis reaction, any specimens that yielded RNA below detection (< 0.25 ng/mL) were spiked into the cDNA synthesis at the maximum possible volume (8 µL).

### Library Preparation and Sequencing

Libraries were prepared from concentrated RNA from each sample spiked with 5 pg of a synthetic RNA construct (ERCC) to serve as an internal control and used the input to the NEBNext Single Cell/Low Input RNA Library Prep Kit (New England Biolabs, Ipswich MA) following manufacturer protocol. Once complete, each sample was quantified with a Quant-iT 1X dsDNA High Sensitivity Assay (Thermo Fisher Scientific, Waltham MA). A sequencing library was set up by combining an equivalent mass from each sample library. Each sequencing library was finally purified with Ampure XP beads (0.9X ratio) (Beckman Coulter, Brea, CA) and quantified with a Qubit 1X dsDNA HS assay (Thermo Fisher Scientific, Waltham MA). Quantified sequencing libraries were diluted, denatured, and sequenced on an Illumina NextSeq 550 using a 150 cycle High Output v2.5 kit following Illumina standard protocols.

### Bioinformatics Methods

Upon completion of sequence generation, all raw FASTQ reads were analyzed through CSI’s *Rapid Active Pathogen Identification & Detection* (RAPID-Dx^®^) bioinformatics pipeline. RAPID-Dx^®^ is an automated bioinformatic workflow that systematically executes the quality filtration, adapter removal, contamination removal, and taxonomy annotation steps within an audit-tracked framework to provide normalized transcriptionally active taxonomy annotation counts within any sample matrix. Briefly, raw data underwent systematic and automated quality filtration and adapter removal using fastp version 0.20.0, in which a sliding window of four bases was trimmed at any point in which average quality dropped below a Q-score of 20 (Chen et al., 2018). Reads trimmed below 75 bases in length were discarded (see **Supplemental Table 1** for individual sample counts). Filtered reads were then processed to remove all *Homo sapiens* contaminant sequences using Kraken2 against the RefSeq *Homo sapiens* reference genome databases with a confidence threshold of 0.4 *via* Kraken2 annotation (Wood et al., 2019). All filtered reads were then subject to systematic parallel annotation, which consists of a two-tiered alignment approach in which filtered reads are first aligned against CSI’s curated clinical genomic database (CGD), which contains full genomes, using a fast k-mer search methodology (Wood et al., 2019). Sequences that are classified as pathogenic taxa of interest above threshold then undergo a subsequent re-annotation process in which the identity of each sequence is confirmed using a local alignment tool, BLAST, to resolve the identity of each pathogenic classification (Camacho et al., 2009). All annotation counts are collated into a dataframe, which contains the observed sequence annotation count for each sample. Post-annotation of transcriptionally active taxa, samples were subject to QC filtration in which samples that yielded less than 1 million internal ERCC control sequences were discarded (**Supplemental Table 2**). A comprehensive overview of metatranscriptome annotation results of NTC specimens from all time points revealed an increased number of raw bacteria and unclassified sequences within the Day 14 NTC (**Supplemental Figure 9**). Additionally, a systematic decrease in ERCC sequences within Day 14 specimens resulted in the entire time point being excluded from all downstream diversity analyses. As a result of QC thresholding, a final count of 105 surface samples were incorporated into all downstream data analyses.

### Alpha and Beta Diversity Analysis of Baseline and Post-Intervention Samples

The generated RAPID-Dx^®^ annotation table as well as all associated sample metadata were merged into a *Phyloseq* version 1.30.0 (McMurdie and Holmes, 2013) object within R 3.6.1 (R Core Team, 2020) for alpha and beta diversity analysis. Annotation counts first underwent internal sequence normalization, in which a per sample quotient of each respective feature by the internal ERCC measure was calculated and then multiplied by a factor of 1,000,000. Observed feature measures were calculated and compared between categorical groups of interest using the Wilcoxon Rank Sum Test and the Holm adjustment using the *rstatix* package version 0.7.0 (Kassambara, 2020). Principal Coordinates Analysis (PCoA) was performed using the *Phyloseq* R package on a weighted Jaccard distance matrix. Significance of PCoA clustering was calculated using the *adonis* test, dispersion was calculated using the *betadisper* test, and unsupervised fitting of taxa to PCoA ordination was generated using *Envfit* within the R package *Vegan* version 2.5-7 (Oksanen et al., 2020)*. Envfit* was run with 999 permutations and species with statistically significant (p<0.001) correlation with the ordination of samples were plotted.

### Microbial Biomarker Analysis of Baseline and Post-Intervention Samples

Kruskal-Wallis tests were conducted to identify significantly differential microbial taxa transcript annotations between samples collected before and after the intervention of the AIONX^®^ cleanSURFACES^®^ (Baseline *vs.* Post) using LEfSe (Segata et al., 2011). Linear Discriminant Analysis (LDA) was used to quantify the strength of enrichment. Enriched taxa transcriptional activity were visualized as a cladogram to show phylogenetic relationships between significantly differential features (Kruskal-Wallis, p ≤ 0.05 and log (LDA)≥1.5). LEfSe analysis was repeated after splitting the data by surface type (Keyboard, OBT, and Work Surfaces), to assess the impact of the AIONX^®^ cleanSURFACES^®^ on both uncovered and covered surfaces.

## Results

**Figure 3:**
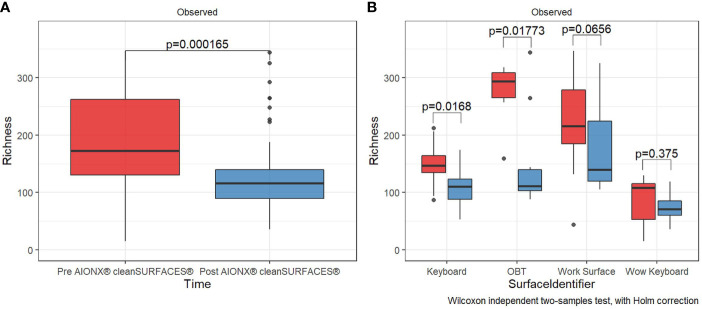
Alpha diversity box plot compares observed taxonomic richness between surface swabs collated before [red] and after [blue] the intervention of AIONX^®^ cleanSURFACES^®^. Overall, a significant decrease [Wilcoxon rank sum Holm adj. p = 0.000165] in transcriptionally active microbial taxa was observed after the intervention of AIONX^®^ cleanSURFACES^®^ **(A)**. When partitioned by surface **(B)**, we observed significant decreases in taxonomic richness after the intervention of the AIONX^®^ cleanSURFACES^®^ on the Keyboard, OBT, and Work Surface [Wilcoxon rank sum Holm adj. p < 0.05].

AIONX^®^ cleanSURFACES^®^ were placed in several patient rooms and nurse stations throughout the ICU (**Figure 1**), with each mat being divided into quadrants so that a different section could be swabbed at each time point (**Figure 2**). Considering all time points (Baseline, Day 1, Day 7, and Day 28) and surfaces (Keyboard, Over-the-bed-table (OBT), Work Surfaces, and Workstation on Wheels, (WOW) Keyboard), a total of 949 million sequences were generated from 191 surface samples, with Post-intervention samples yielding significantly fewer filtered microbial sequences (Wilcoxon, p=0.019). Bioinformatic ribosomal RNA (rRNA) annotation revealed a range of 141 – 51,820 sequences annotated as rRNA within experimental surface samples. After quality filtration and removal of controls, a total of 105 samples were normalized and used for downstream analysis. A summary of sequencing QC results can be found in **Supplemental Table 1**. Microbial community richness was significantly (Kruskal-Wallis, p<0.001) depleted Post cleanSURFACES^®^ application (**Figure 3A**). This trend of depleted richness held for all surfaces (**Figure 3B**) and time points (**Supplemental Figure 1**). Baseline samples had an average of 184.5 unique microbial taxa, with a standard error of 13.3, and Post samples only had an average of 129.9 microbial taxa, with a standard error of 8.2. Considering the different surfaces, richness was lower (P < 0.05) for all Post cleanSURFACES^®^ intervention samples and significantly (P < 0.05) lower for Keyboard and OBT surfaces (**Figures 3A, B**). Therefore, samples from surfaces after cleanSURFACES^®^ antimicrobial mat application yielded roughly 30% fewer unique microbial species (presence or absence) globally, though the cleanSURFACES^®^ intervention seems to have been most effective for Keyboard and OBT surfaces.

**Figure 4:**
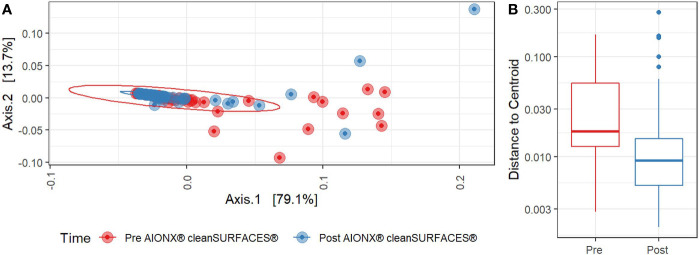
**(A)** Principal Coordinates Analysis (PCoA) plot of weighted Jaccard distances reveals significant differences [PERMANOVA, p=0.005] in global microbial transcriptional profiles between samples collected before (Red) and after (Blue) the intervention of the AIONX^®^ cleanSURFACES^®^. **(B)**. Boxplot comparing within-group dispersion between samples collected before (Red) and after (Blue) AIONX^®^ cleanSURFACE^®^ intervention.

Both Baseline and Post-intervention samples were generally clustered in the same area of the PCoA plot (**Figure 4A**), but the clustering distribution was much tighter for Post samples (**Figure 4B**). Moreover, beta diversity was significantly distinct before and after cleanSURFACES^®^ intervention (PERMANOVA, p=0.006). This shows that microbial communities in Post samples tended to be more similar to each other as compared to communities from the Baseline samples. Considering different surfaces, Keyboard and OBT had noticeably tighter clustering in Post samples, while Work Surfaces (WS) and WOW Keyboards had more similar dispersion levels between Baseline and Post-intervention samples (**Supplemental Figure 2**). When all surfaces were grouped by timepoint, all three Post application time points had tighter clustering and lower median distances among samples compared to Baseline samples (**Supplemental Figure 3**). Unsupervised *Envfit* analysis identified three species (*Staphylococcus epidermidis*, *Staphylococcus hominis*, and *Malassezia restricta*) that significantly (P <0.001) contributed to variation explained in PCoA ordination’s first axis (**Supplemental Figure 4**).

**Figure 5:**
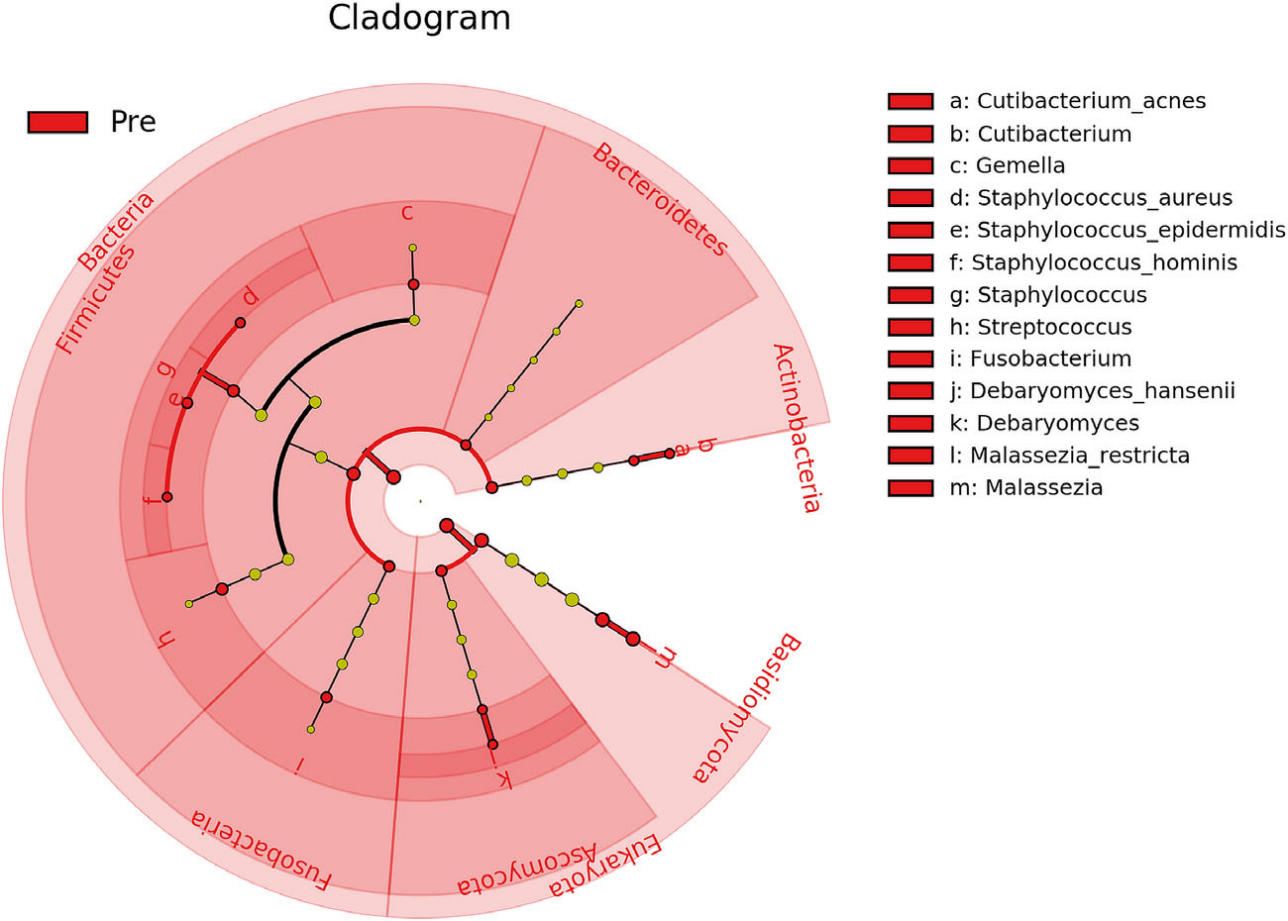
Cladogram of differential taxa identified between samples collected before (Pre/Baseline) and after (Post) the intervention of AIONX^®^ cleanSURFACES^®^. Taxa that were enriched in Pre/Baseline are indicated with red nodes, while taxa that were enriched in the Post intervention samples are indicated with blue nodes. Significant (Kruskal-Wallis, p ≤ 0.05 and log (LDA)≥1.5) features are shown and are composed of 21 enriched taxa within the Baseline intervention group. Notably, the Bacteria as a domain were significantly reduced in samples Post-AIONX^®^ intervention. No enriched microbial features were observed within the Post-AIONX^®^ intervention group.

Furthermore, biomarker analysis revealed 21 microbial taxa (domains, phyla, genera, and species) that differed significantly (P ≤ 0.05, log (LDA)≥1.5) between Baseline and Post-intervention samples (**Figure 5**), with all 21 being enriched in Baseline. Therefore, both Bacteria and Fungi were less active in samples collected after cleanSURFACES^®^ mat application. Within those domains, the bacterial phyla *Actinobacteria*, *Bacteroidetes*, *Firmicutes*, and *Fusobacteria* and the eukaryotic phyla *Ascomycota* and *Basidiomycota* exhibited significantly (P ≤ 0.05, log (LDA)≥1.5) depleted transcript annotations Post cleanSURFACES^®^ intervention. Multiple taxa within those phyla were identified as significantly differential as well. Specifically, *Cutibacterium, Gemella*, *Staphylococcus*, *Streptococcus*, *Fusobacterium*, *Debaryomyces*, and *Malassezia* were reduced in Post samples.

However, as shown by the alpha diversity analysis, microbial communities across surfaces were not all impacted to the same extent (**Supplemental Figure 5**). Baseline OBT surfaces exclusively enriched 33 taxa, implying that transcripts for these taxa were depleted during the Post cleanSURFACES^®^ intervention. The Keyboard and Work Surfaces (WS) samples had a mix of taxa enriched in Baseline and Post groups, with both having more taxa depleted Post cleanSURFACES^®^ intervention. Contrastingly, WOW Keyboard had no transcripts for taxa enriched in Baseline or Post groups. Pairwise time point comparisons revealed Baseline to Day 1 had the domain, Bacteria, significantly reduced, as well as three bacterial and one fungal phyla (**Supplemental Figure 6**). Day 7 had four bacterial and two fungal phyla depleted, but *Proteobacteria* was enriched in Day 7 compared to Baseline (**Supplemental Figure 7**). Similar to Day 1, Day 28 had Bacteria depleted, including five bacterial phyla, and *Basidiomycota* reduced as well (**Supplemental Figure 8**). Across all time points, the Keyboard surface had the greatest number of taxa (Domains, Phyla, Genera, and Species) depleted compared to Baseline (n=44).

**Figure 6:**
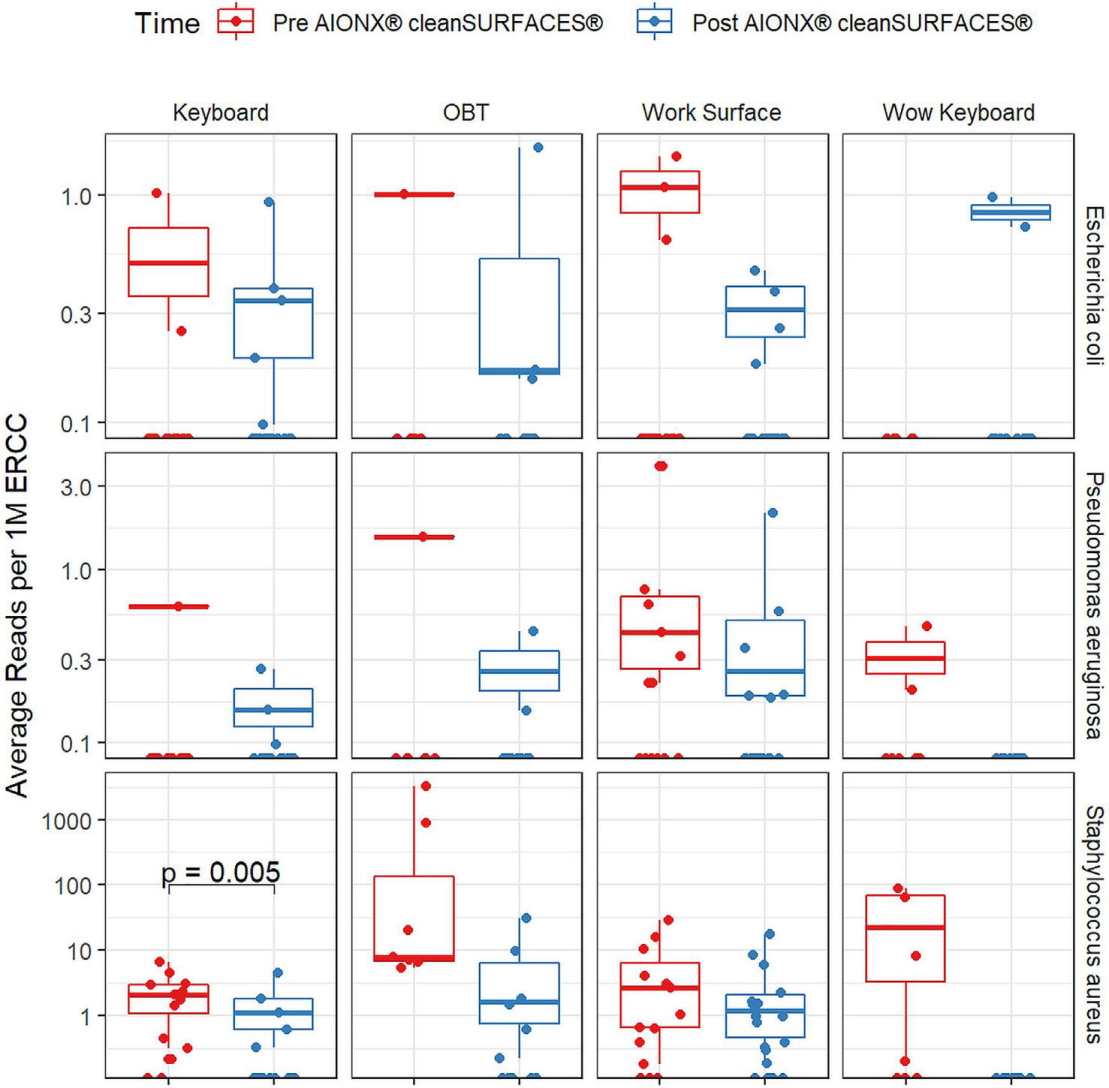
Normalized average transcript count comparison boxplot of “Common Pathogens” between surfaces before and after the intervention of the AIONX^®^ cleanSURFACES^®^. Statistically significant (adjusted p < 0.05) comparisons are marked with an asterisk.

**Figure 7:**
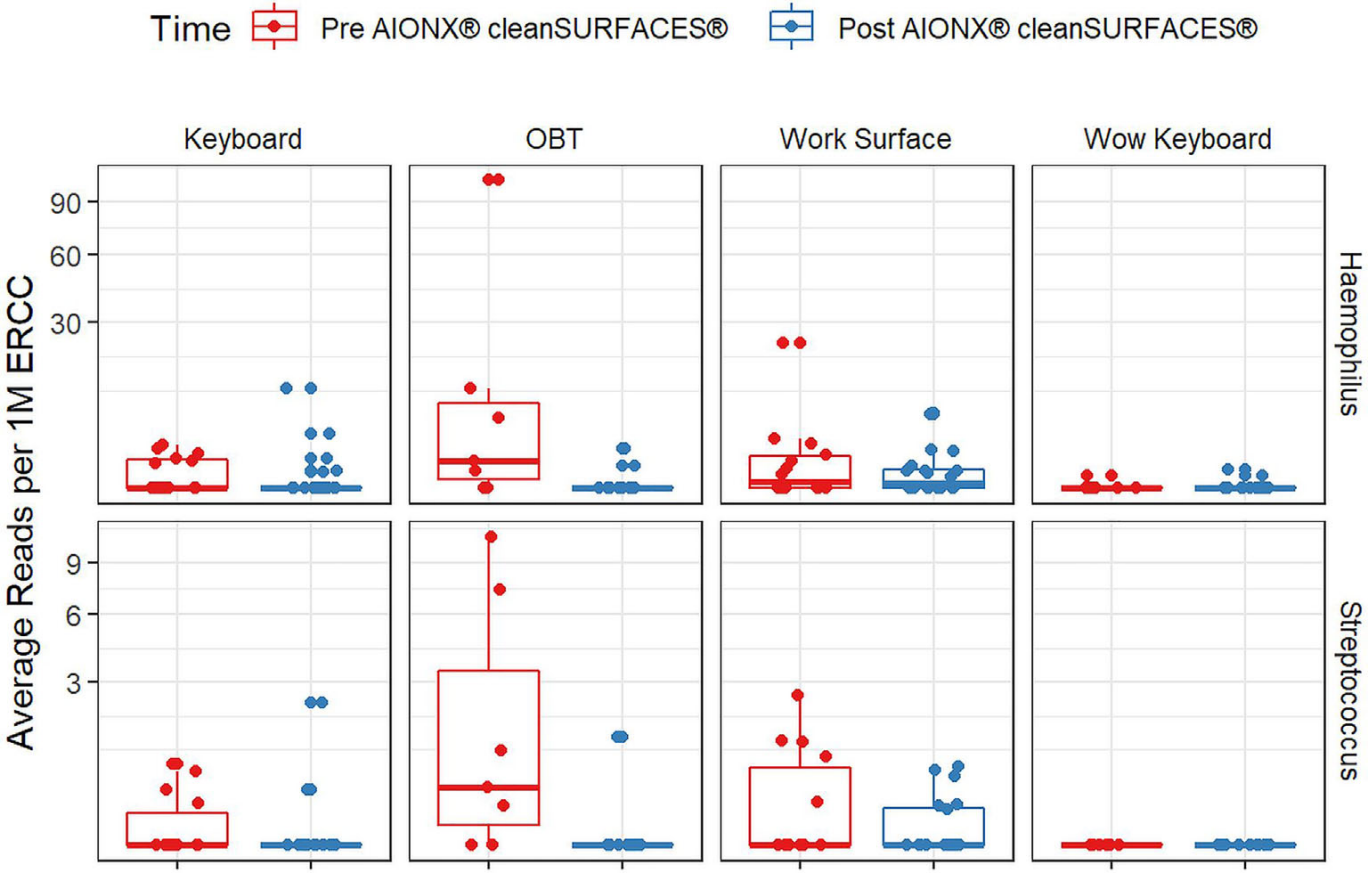
Normalized average transcript count comparison boxplot of “Respiratory Pathogens’’ between surfaces before and after the intervention of the AIONX^®^ cleanSURFACES^®^. Statistically significant (adjusted p < 0.05) comparisons are marked with an asterisk.

**Figure 8:**
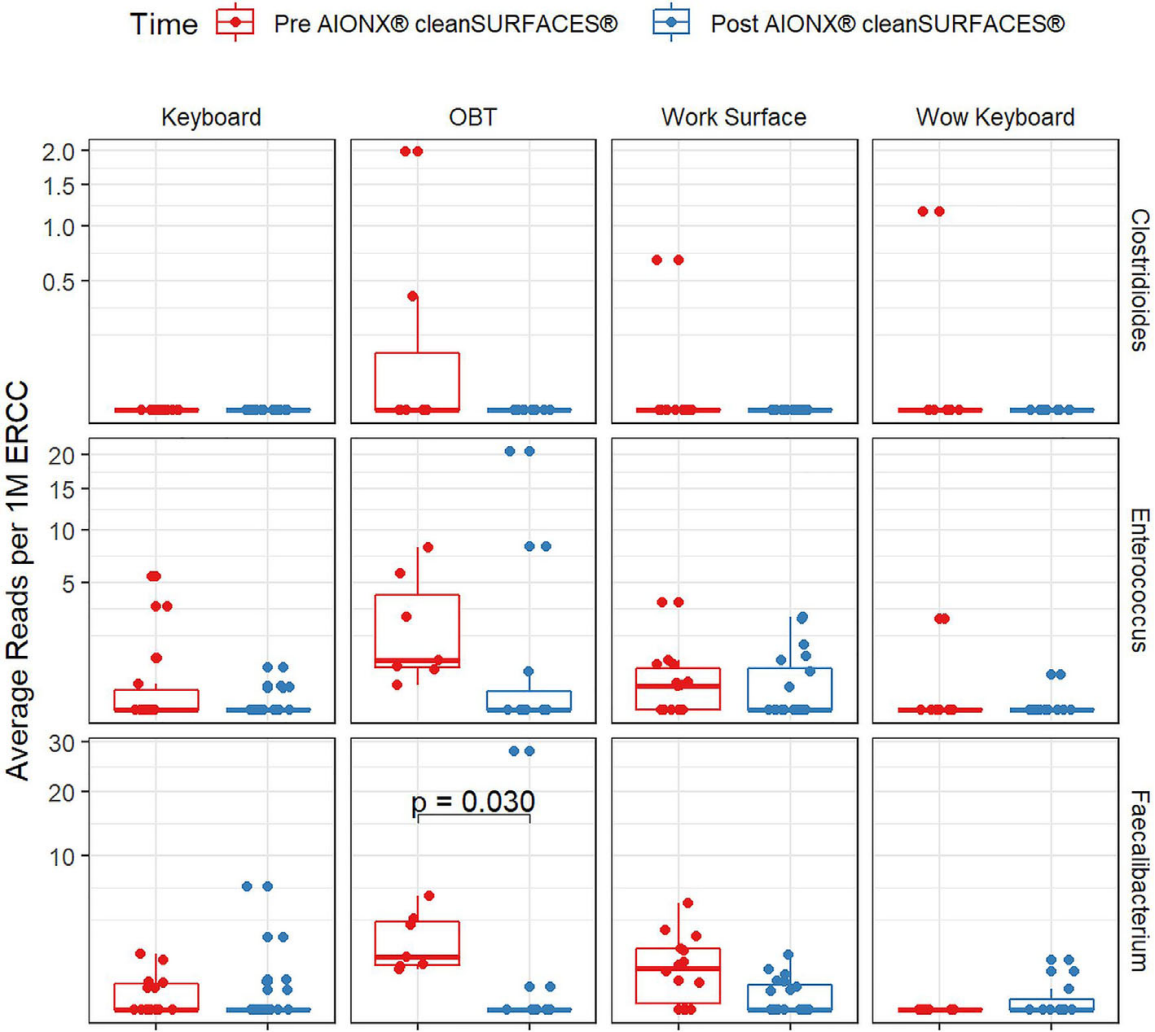
Normalized average transcript count comparison boxplot of fecal biomarker taxa between surfaces before and after the intervention of the AIONX^®^ cleanSURFACES^®^. Statistically significant (adjusted p < 0.05) comparisons are marked with an asterisk.

Importantly, common opportunistic pathogens were also depleted Post cleanSURFACES^®^ intervention (**Figure 6**). While transcript annotations for *Escherichia coli*, *Pseudomonas aeruginosa*, and *S. aureus* were all reduced on Keyboard, OBT, and Work Surfaces following the cleanSURFACES^®^ intervention, reduction of *S. aureus* transcripts was the only statistically significant reduction observed (P = 0.005). With respect to potential respiratory pathogenic genera (*Haemophilus* and *Streptococcus)*, there were no significant reduction in transcripts observed after application of cleanSURFACES^®^ intervention (**Figure 7**) (LaRocque and Ryan, 2019). Transcripts for the fecal biomarker taxa, *Faecalibacterium*, were significantly (P = 0.03) depleted on OBT surfaces (Sehgal and Khanna, 2021). While observed reductions from Baseline to Post-intervention are not statistically significant for *Enterococcus* and *Clostridioides*, transcripts were shown to be depleted on OBT surfaces (**Figure 8**). Additional analysis revealed average transcript counts of select biomarker taxa decreased from Baseline (hour 4, 8) to Day 7 (**Supplemental Figure 10**). However, by Day 28, there is an increase in average transcript counts of several biomarker taxa (*Enterococcus faecalis*, *Haemophilus parainfluenzae*, and *S. aureus*) on keyboards and WS, and OBT surfaces (**Supplemental Figure 10**). Notably, Baseline samples collected at hours 0 and 1 were filtered out based on existing QC thresholds and were subsequently not included in any downstream analysis.

## Discussion

Recent work evaluating disinfection efficacy has largely focused on the impact of terminal cleaning strategies through culture-based methods. Amongst this literature, several studies assessed the efficacy of conventional disinfection sprays, wipes, and UV disinfection. These studies report a variability in efficacy (Boyce et al., 2011; Manian et al., 2011; Shaughnessy et al., 2013; Koscova et al., 2018), and suffer from limitations associated with culture-based assays (Hiergeist et al., 2015; Shrestha et al., 2015). Consequently, we employed a novel metatranscriptomics-based pathogen detection pipeline, CSI-Dx™, to evaluate the effectiveness of a continuous-cleaning product, cleanSURFACES^®^, with a focus on its potential in reduction of active pathogens in an intensive care unit (ICU) environment. Importantly, this pipeline allowed us to characterize all active microbes, including fungi and viruses, both of which would have been undetectable using a 16S rRNA gene amplicon approach due to lacking that gene.

### Alpha and Beta Diversity Differences Between Several Baseline and Post-Intervention Surfaces

Our study revealed a significant decrease (P < 0.05) in observed microbial richness from Baseline to Post-intervention samples (**Figure 3**). In particular, two surface types (OBT and WS) exhibited the most significant differences (P < 0.05) in richness from Baseline to Post-intervention (**Figure 2**). The same trend of decreased observed richness also holds when individual time points (Day 1, Day 7, and Day 28) are compared to Baseline (**Supplemental Figure 1**). After cleanSURFACES^®^ intervention, there is a global reduction of ~30% in the number of unique microbial species detected, which is concordant with previous terminal cleaning literature (Attaway et al., 2012; Schmidt et al., 2016; Koscova et al., 2018; Schmidt et al., 2019).

Microbial community composition between Baseline and Post-intervention groups were found to be significantly distinct (PERMANOVA, P = 0.006; **Figure 4**). These differences can also be observed over time where Baseline microbial communities exhibited a wider degree of dispersion compared to Post-samples (**Supplemental Figure 3**). Of the three species (*S. epidermidis*, *S. hominis*, and *M. restricta*) found to significantly (P < 0.001) contribute to the degree of explained variance (**Supplemental Figure 4**), *S. epidermidis* and *S. hominis* can cause nosocomial infections (Palazzo et al., 2008) though *S. epidermis* is also a common skin commensal bacteria (Byrd et al., 2018). These results highlight the potential for NGS technology in detecting nosocomial pathogens at high taxonomic resolution from ICU environmental surfaces. Interestingly, others have also observed similar microbes to colonize subjects within ICU wards. For instance, *Staphylococcus* spp., and *Streptococcus* spp. have been detected in the noses of babies after NICU hospitalization using 16S rRNA gene amplicon sequencing (Cason et al., 2021). Including the study by Cason et al., findings from recent studies suggest that environmental hospital surface contamination (i.e. *Staphylococcus* spp.) can greatly contribute to the transmission of nosocomial pathogens and the occurrence of HAIs (Suleyman et al., 2018; Cason et al., 2021). While this study reports on the microbial community composition of active microbes from ICU surfaces Pre and Post cleanSURFACES^®^ intervention, more work is required to assess the potential impact of cleanSURFACES^®^ on the transmission of nosocomial pathogens and HAI occurrence.

Overall, these findings suggest that daily chemical disinfection methods, in conjunction with cleanSURFACES^®^ technology is not only reducing transcript annotations for certain microbes that were previously detected but also continuously maintaining a distinct and less diverse microbial community composition compared to Baseline, even at 28 days after cleanSURFACES^®^ installation. The necessity to maintain decontaminated surfaces over time has been demonstrated through studies where re-contamination was reported from 2.5 hrs to 24 hrs after disinfection events (Scott et al., 1984; Attaway et al., 2012; Stewart et al., 2014). Results from our study demonstrate that the application of cleanSURFACES^®^ technology in union with periodic cleaning protocols effectively combats against the re-contamination of several key pathogenic taxa initially observed at the baseline timepoint, during which only terminal cleaning protocols were utilized.

### Reduction of Clinically Relevant Pathogens

Transcript annotations of several clinically relevant pathogens were depleted on multiple surfaces included in this study, further demonstrating the effectiveness of cleanSURFACES^®^ (**Figures 6**–**8**, **Supplemental Figures 5** and **10**). In addition to other common opportunistic pathogens detected, transcript annotations of *S. aureus* were significantly depleted (P = 0.005) on Keyboard surfaces following the cleanSURFACES^®^ intervention (**Figure 6**). While not statistically significant, transcripts of the genus *Clostridioides*, which consists of *Clostridium difficile*, an opportunistic pathogen associated with infectious diarrhea (Guh and Kutty, 2018), were depleted on Post-intervention OBT surfaces (**Figure 8**). A multitude of studies have highlighted the challenge of eliminating certain opportunistic pathogens (including *S. aureus*, *C. difficile*, methicillin-resistant *S. aureus*), which are recalcitrant to conventional terminal cleaning methods (French et al., 2004; Weinstein and Hota, 2004; Siani et al., 2011; Fagerlund et al., 2017; Haak and Wiersinga, 2020). While we cannot comment on methicillin-resistant *S. aureus*, the species *S. aureus* was significantly (P ≤ 0.05, log (LDA)≥1.5) less active in Post-intervention samples compared to Baseline, while *C. difficile* did not differ significantly between Baseline and Post-intervention (**Figure 5**). Among other key respiratory and fecal-associated pathogens accounted for, transcript annotations for the opportunistic pathogen, *Faecalibacterium*, was also shown to be significantly depleted (P=0.03) on several surfaces in our study (**Figure 8**). Additional analysis of selected biomarker taxa over time suggests that the average transcripts of certain opportunistic pathogens (*E. faecalis*, *H. parainfluenzae*, and *S. aureus*) are decreasing from Baseline to Day 7 of the intervention. However, by Day 28 these taxa have higher transcript counts across most surfaces (except WOW keyboards). Overall, these results suggest that daily chemical disinfection methods used jointly with cleanSURFACES^®^ are effectively reducing a number of clinically relevant opportunistic pathogens across different surfaces. However, issues surrounding the longevity of cleanSURFACES^®^ technology were observed through the increase of transcript annotations of taxa on surfaces by Day 28.

### Differential Taxa Enrichment Between Baseline and Post-Intervention Surfaces

Baseline and Post-intervention comparisons revealed 21 microbial taxa (domains, phyla, genera, and species) to be significantly (P ≤ 0.05, log (LDA)≥1.5) depleted in Post-intervention samples (**Figure 5**). These taxa have been previously identified in a variety of hospital settings through culture dependent and independent methods (Bokulich et al., 2013; Comar et al., 2019; Haak and Wiersinga, 2020). One study characterized antimicrobial resistance within a hospital and largely found skin-associated microbes on surfaces, including *Cutibacterium acnes* and *S. epidermidis* (Haak and Wiersinga, 2020). While both are common skin microbes, they also have been implicated in HAIs associated with surgical wounds (Ziebuhr et al., 2006; Otto, 2009; Elston et al., 2019). Prior to routine cleaning in a NICU, elevated *Streptococcus* spp., *Staphylococcus* spp., and *Gemella*, were also found on high-contact surfaces (Bokulich et al., 2013), and these microbes have been reported to pose a threat to health ranging from mild to severe infections (La Scola and Raoult, 1998; Gray and Stevens, 2009; Zanger et al., 2010; Leung et al., 2011). In other cases, certain *Gemella* spp. have been revealed to be opportunistic respiratory pathogens (La Scola and Raoult, 1998; Jayananda et al., 2017). Among the fungi that were enriched in Baseline samples, some were found to be commensal skin flora (*Malassezia* spp.), whereas *Debaryomyces hansenii* has been shown to be a rare human fungal opportunistic pathogen (Desnos-Ollivier et al., 2008; Ianiri et al., 2018). More recently, several *Malassezia* spp. have been associated with mild to moderate skin conditions (Saunders et al., 2012). Overall, biomarker analysis suggests that daily chemical disinfection methods used with cleanSURFACE^®^ mats are successfully reducing transcript annotations of potentially life-threatening opportunistic pathogens that were previously observed in Baseline samples, where only terminal cleaning protocols were utilized.

Biomarker analysis performed on each surface type yielded similar results, in which opportunistic pathogens were enriched among Baseline samples (**Supplemental Figure 5**). Both Baseline keyboard and WS exhibited an enrichment of opportunistic pathogens (i.e., *Staphylococcus* spp.*, Streptococcus* spp.*, Gemella*, and *Debaryomyces* spp.) in addition to human skin associated microbes. However, keyboards had an enrichment of more opportunistic pathogens compared to WS, including *Enterococcus faecium, Halomonas* spp., *Dialaster* spp., *Finegoldia* spp., and *Fusarium oxysporum* (Eljaschewitsch et al., 1996; Morio et al., 2007; Stevens et al., 2009; Brodrick et al., 2016; Neumann et al., 2020). This increase of unique, enriched opportunistic pathogens on keyboards are to be expected as a result of challenges associated with disinfecting keyboards compared to flat work surfaces. Additionally, Baseline OBT samples were found to enrich for pathogens responsible for a multitude of diseases; from mouth/gum diseases and hand infections, to septic arthritis (Arons et al., 1982; Wu et al., 1992; Baghban and Gupta, 2016; Mashima et al., 2016; Vartoukian et al., 2016). It is reasonable to observe an enrichment of oral-derived microbes on OBT since patients are likely using these tables to eat and drink while in bed. While the species of *Neisseria* enriched from Baseline OBT samples remain unknown, two species are recognized to be life-threatening, *N. meningitidis* and *N. gonorrhoeae* (Tzeng and Stephens, 2000; Unemo and Shafer, 2014).

Post-intervention surfaces were largely dominated by innocuous organisms like *Sphingobium* spp., *Sphingomonas* spp., and *Rhodopseudomonas* spp., *Acinetobacter* spp., *Delftia* spp. (environmentally-derived bacteria) in addition to commensal human gut (*Ruminococcus*) and vaginal bacteria (*Lactobacillus iners*; **Supplemental Figure 5**) (Huang et al., 2009; Verma et al., 2014; Petrova et al., 2017; La Reau and Suen, 2018). However, Post-intervention keyboard and WS surfaces enriched for potential opportunistic pathogens as well, such as *Acinetobacter baumannii* and *Corynebacteria* spp., respectively (**Supplemental Figures 5, 7**) (Peleg et al., 2008; Zasada and Mosiej, 2018). More specifically, both Baseline and Post-intervention WS enriched for *Corynebacterium* and *Corynebacterium* spp. NML98_0116 (**Supplemental Figure 7**). While *Corynebacteria* is a common skin commensal, non-diphtheritic *Corynebacteria* has become an emerging nosocomial opportunistic pathogen among immunocompromised individuals (Rudresh et al., 2015). While these findings imply that joint application of daily chemical disinfection practices with cleanSURFACES^®^ technology may not be eliminating all possible opportunistic pathogens from high-contact surfaces, there is also a distinct increase in diversity of innocuous microorganisms largely found in the environment (water and soils). Increases of these microbial taxa on surfaces could be a result of variations in building design/operation (source and rate of outdoor air ventilation), shedding from building inhabitants, and/or variations in terminal cleaning methods between housekeeping (Hospodsky et al., 2012; Kembel et al., 2012; Hewitt et al., 2013; Stephens, 2016; National Academies of Sciences et al., 2017). However, these factors likely did not change as a result of the application of the mats, as the mats were installed into the existing hospital environment. Therefore, their use would not be expected to change building operation or increase how often the surfaces were exposed to shedding from inhabitants, and as previously stated, staff were specifically instructed not to change their cleaning methods.

### Limitations and Challenges

Day 7 and 28 time points revealed all surfaces (except for WOW keyboards) exhibited varying degrees of enrichment for environmentally related microbes (**Supplemental Figures 6–8**). In particular, Day 7 samples demonstrated the largest amount of significantly enriched environmentally related, harmless microbes compared to day 1 and 28. However, by day 28, there is a considerable decrease in diversity of enriched environmentally related microbes compared to surfaces from Day 7. These results suggest that the efficiency of cleanSURFACES^®^ mats in reducing clinically relevant opportunistic pathogens reach a peak around 7 days after initial implementation and, at 28 days past application, there may be a decrease in efficacy occurring. As a consequence of repeated sanitation with chemical disinfection wipes throughout the sampling period, there were several non-functional cleanSURFACES^®^ mats by Day 28 of the intervention. The occurrence of non-functional mats by the end of the study suggests potential issues relating to the longevity of this version of the technology. The increase of these microbes on surfaces could also be a result of variations in building design/operation (source and rate of outdoor air ventilation), shedding from building inhabitants, and/or variation in terminal cleaning methods between housekeeping (Hospodsky et al., 2012; Kembel et al., 2012; Hewitt et al., 2013; Stephens, 2016; National Academies of Sciences et al., 2017). While building conditions and inhabitants can impact the microbial community profiles reported in our study, it remains important to note that the presence of the mats themselves may have caused behavioral changes in the patients and nurses, which could have contributed to taxonomic shifts observed. Still, cleanSURFACES^®^ mats were likely the predominant driver of these shifts, as there was a consistent reduction in alpha diversity among all surface types and staff were instructed not to change their cleaning behaviors. Although temporal fluctuations of microbes shed by patients and staff could have impacted our results, it remains unlikely since observed shifts in taxa between Post-intervention samples are consistent throughout duration of the study.

Unlike other surfaces included in this study, there were no significantly enriched transcripts for taxa between Baseline and Post-intervention WOW keyboards (**Supplemental Figure 5**). The lack of enriched transcripts for taxa can be due to ineffective placement of cleanSURFACES^®^ mats in relation to WOW keyboards. For instance, mats could have been placed in a position that nurses did not regularly touch before using the keyboard on WOW surfaces. In this situation, cleanSURFACES^®^ mats were placed underneath the monitor of the WOW unit, since WOW keyboards are confined inside drawers. The significance of proper exposure to surfaces for disinfection has been highlighted in a similar efficacy study utilizing a mobile UV light unit (Boyce et al., 2011). In the case of our study, the placement and orientation of the cleanSURFACES^®^ mat in relation to keyboards on WOW surfaces could have contributed to the minimal elimination of contamination on this specific surface. Keyboard surfaces could have exhibited more significant reductions in richness compared to WOW keyboards due to the direct placement of cleanSURFACES^®^ mats below the keyboard, as shown in **Figure 2**.

Apart from the Baseline samples collected prior to the cleanSURFACES^®^ intervention, no additional control samples were collected in parallel with Post-intervention samples. The lack of controls collected in parallel to Post-intervention samples serve as an additional limitation to the overall study. However, a true control at each subsequent time point is difficult to create since the presence of a functional cleanSURFACES^®^ mat in the ICU can potentially create bias and impact the transmission network of microbes from surface to surface. In future studies to circumvent this limitation, it may be beneficial to collect simultaneous samples from an ICU with the intervention and from another ICU in the same hospital without the intervention. In the case of this study, UPMC Harrisburg did not have an additional equivalent ICU with similar patient population and disease severity index.

Lastly, no functional annotation was performed, as the focus of this work was solely to identify shifts in active taxa over time in concordance with the cleanSURFACES^®^ mat application. However, future work could focus on functional annotations to provide useful insight into changing expression of antibiotic resistance genes, virulence factors, and other important microbial metabolic pathways.

## Conclusions

In this study, we assessed the efficacy of a continuous cleaning technology, cleanSURFACES^®^ in a UPMC intensive care unit over time, using a metatranscriptomics sequencing approach. To our knowledge, this is the first study to apply a metatranscriptomics technology to characterize active microbial communities on hospital surfaces in response to an application of a continuous cleaning technology. Baseline results demonstrated that microbial communities on high-contact surfaces largely consisted of opportunistic pathogens and skin commensals. These results are consistent with findings reported by previous studies utilizing culture-dependent and independent methods to characterize hospital microbial communities (Bokulich et al., 2013; Comar et al., 2019; Haak and Wiersinga, 2020). Overarching findings from this study demonstrate that cleanSURFACES^®^ could prove to be a promising technology that complements the current chemical disinfection methods used at UPMC Harrisburg in reducing surface contamination. While the data from this study highlight the efficacy of daily chemical disinfection methods in conjunction with cleanSURFACES^®^ in reducing the transcriptional activity of several clinically important microbes, future work should also include evaluation of cleanSURFACES^®^ in multi-center studies in concert with tracking HAI incidence. Additional analyses including functional gene classification and comparisons will be assessed in future multi-center investigations.

## Data Availability Statement

The datasets presented in this study can be found in online repositories. The names of the repository/repositories and accession number(s) can be found below: https://www.ncbi.nlm.nih.gov/, PRJNA703385.

## Author Contributions

AS, AW, SK, JG, and RL conceived and designed the experiments. JB collected samples. TL, SA, and CM processed the samples for sequencing. JC, CB, VT, and JW analyzed the data. JC, TL, AS, SK, JG, JW, and RL wrote and revised the manuscript. All authors contributed to the article and approved the submitted version.

## Funding

This material is based on research sponsored by Air Force Research Laboratory under Agreement Number FA8650-20-2-5506 in support of Air Force Research Laboratory. The U.S. Government is authorized to reproduce and distribute reprints for Governmental purposes notwithstanding any copyright notation thereon. The views and conclusions contained herein are those of the authors and should not be interpreted as necessarily representing the official policies or endorsements, either expressed or implied, of Air Force Research Laboratory, the Air Force Research Laboratory or the U.S. Government.

## Conflict of Interest

JC, TL, SA, CM, CB and VT were employed by Contamination Source Identification, LLC. RL, JW, and AS are owners of Contamination Source Identification, LLC. JB was employed by AIONX^®^. AS served as a consultant for AIONX^®^. AW was employed by Nextflex.

The remaining authors declare that the research was conducted in the absence of any commercial or financial relationships that could be construed as a potential conflict of interest.
