# Supplementary material for: A Metatranscriptomics Survey of Microbial Diversity on Surfaces Post-Intervention of cleanSURFACES® Technology in an Intensive Care Unit
Source: Front Cell Infect Microbiol. 2021 Jul 20;11:705593. doi: 10.3389/fcimb.2021.705593 (PMC8330600; doi:10.3389/fcimb.2021.705593)
Supplement: Supplementary file 1 [file DataSheet_1.docx]

Supplementary Material


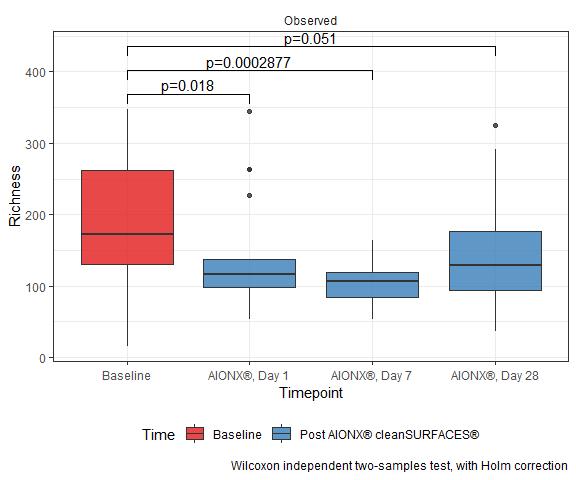


**Supplemental Figure 1.** Alpha diversity box plot compares observed taxonomic richness between surface swabs collated before [red] and after [blue] the intervention of AIONX® cleanSURFACES® stratified by sampling day.


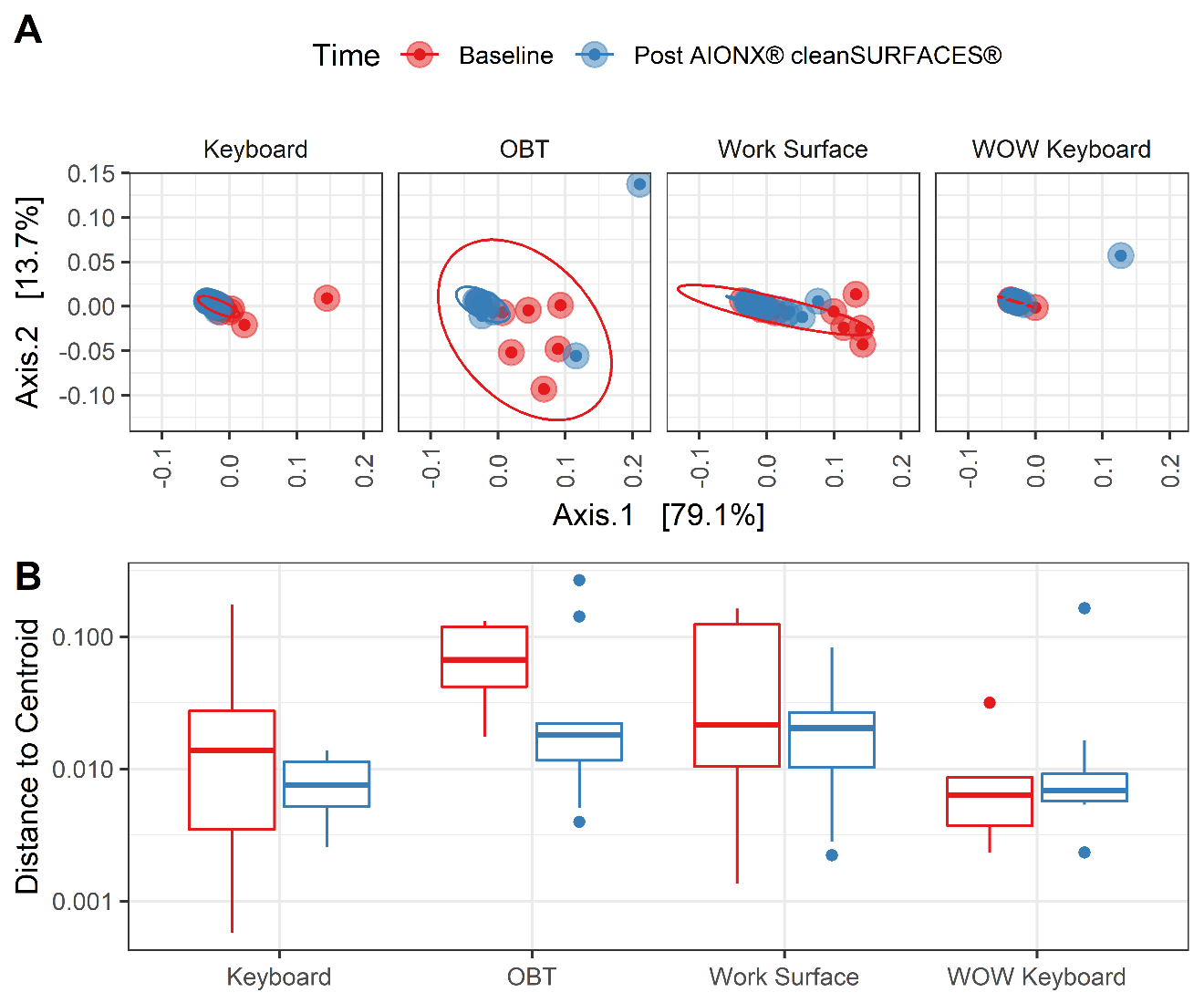


**Supplemental Figure 2.** (A) Principal Coordinates Analysis (PCoA) plot of weighted Jaccard distances reveals differences in global microbial transcriptional profiles between samples collected before (Red) and after (Blue) the intervention of the AIONX® cleanSURFACES® stratified by surface. (B). Boxplot comparing within-group dispersion between samples collected before (Red) and after (Blue) AIONX® cleanSURFACE® intervention stratified by surface.


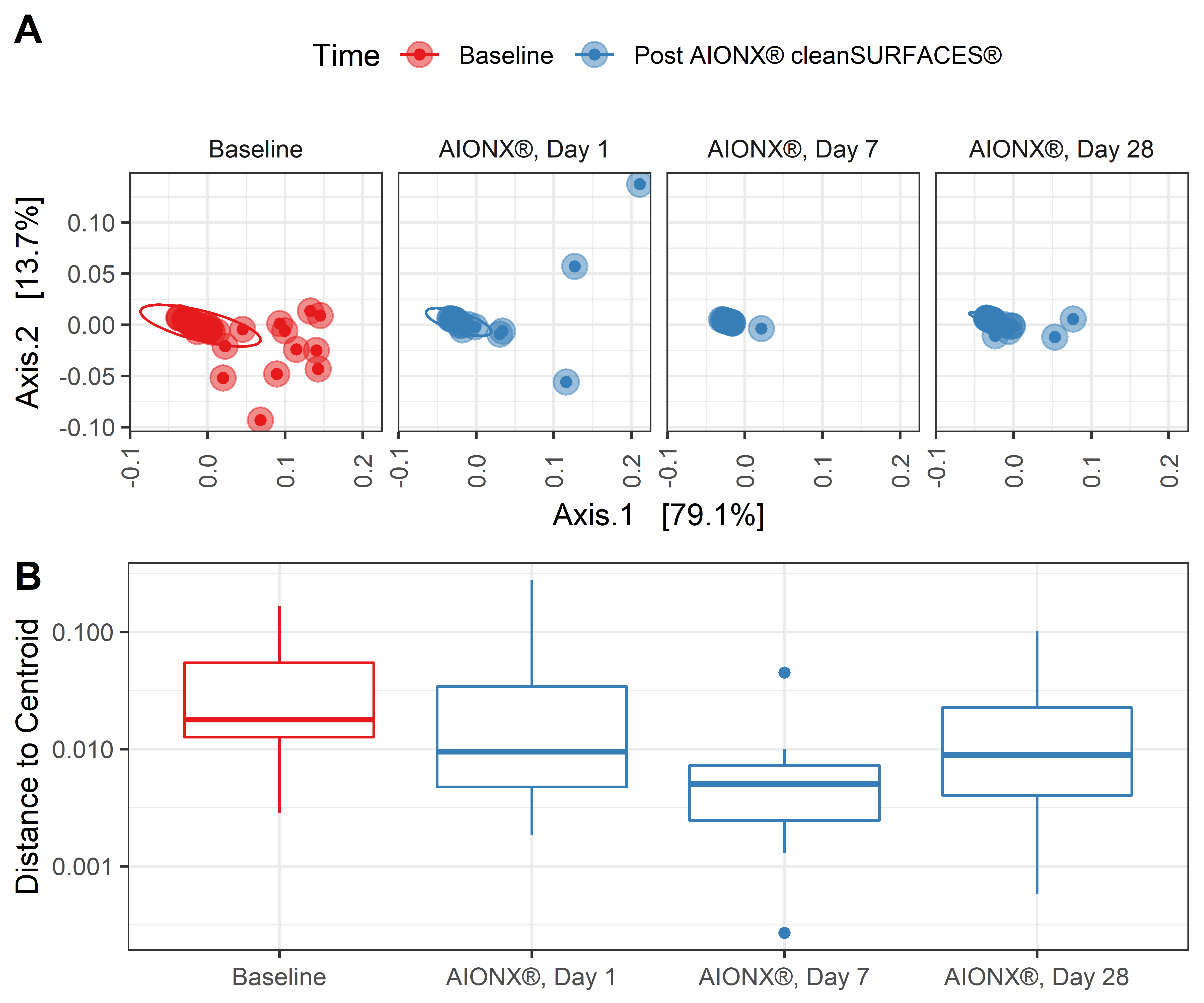


**Supplemental Figure 3.** (A) Principal Coordinates Analysis (PCoA) plot of weighted Jaccard distances reveals significant differences in global microbial transcriptional profiles between samples collected before (Red) and after (Blue) the intervention of the AIONX® cleanSURFACES® stratified by sampling day. (B). Boxplot comparing within-group dispersion between samples collected before (Red) and after (Blue) AIONX® cleanSURFACE® intervention stratified by sampling day.

**
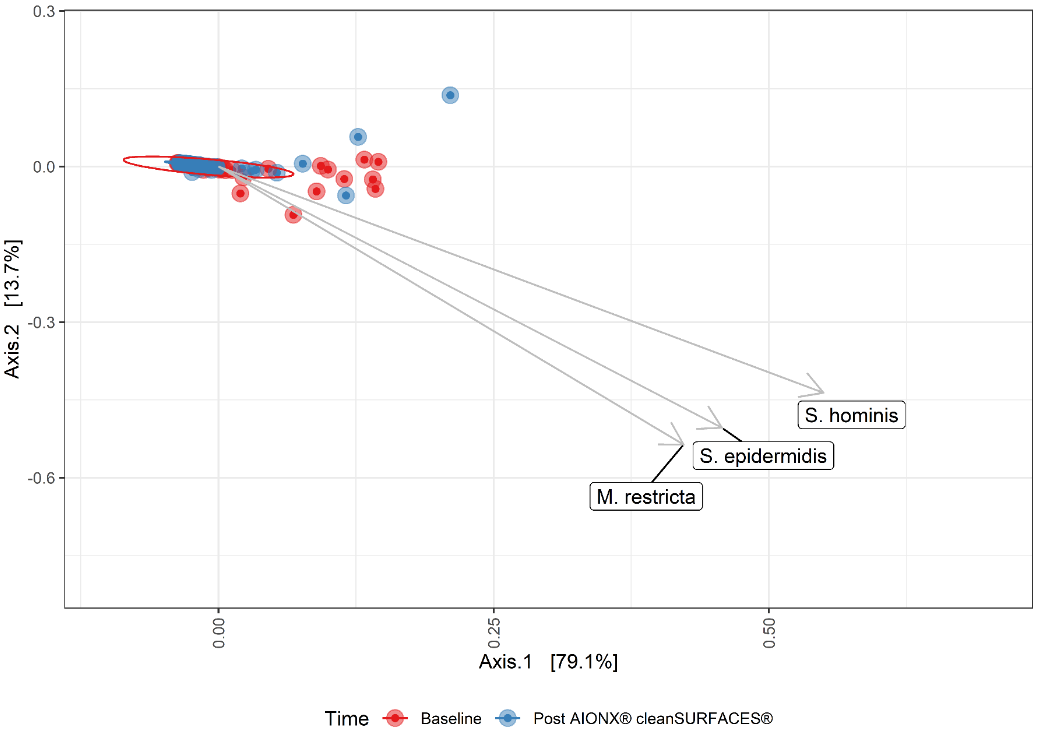
**

**Supplemental Figure 4.** Principal Coordinate Analysis (PCoA) plot of weighted Jaccard distances with Envfit vector analysis and overlay reveals differential clustering of surface samples collected before and after the intervention of the AIONX® cleanSURFACES®. Using unsupervised Envfit analysis from the R package Vegan, three species were identified and fitted to the ordination. These taxa were found to significantly contribute to the percent of explained variance along axis 1 (p-value < 0.001).


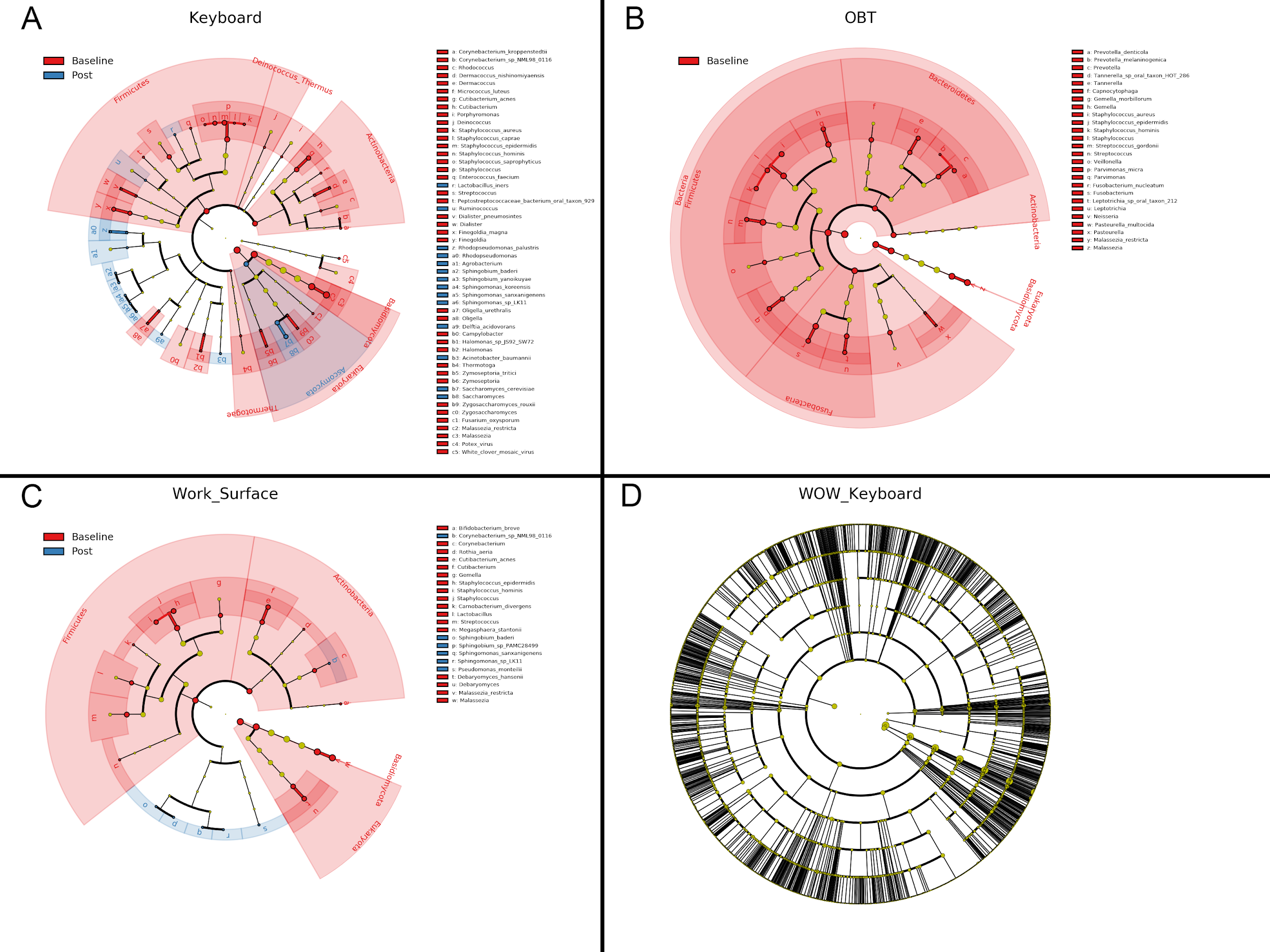


**Supplemental Figure 5.** LEfSe cladograms of differential baseline vs. Post taxa (domain, phylum, genus, and species) by surface: (A) Keyboard, (B) OBT, (C) Work Surfaces, (D) WOW Keyboard. All surfaces had more taxa enriched in baseline, except for WOW Keyboard, which had no significantly differential taxa. OBT’s comparison (B) revealed a lack of enriched taxa for the Post group. Keyboard (A) and Work Surfaces (C) had taxa enriched in both baseline and Post. However, both surfaces only had enriched phyla in the baseline group.


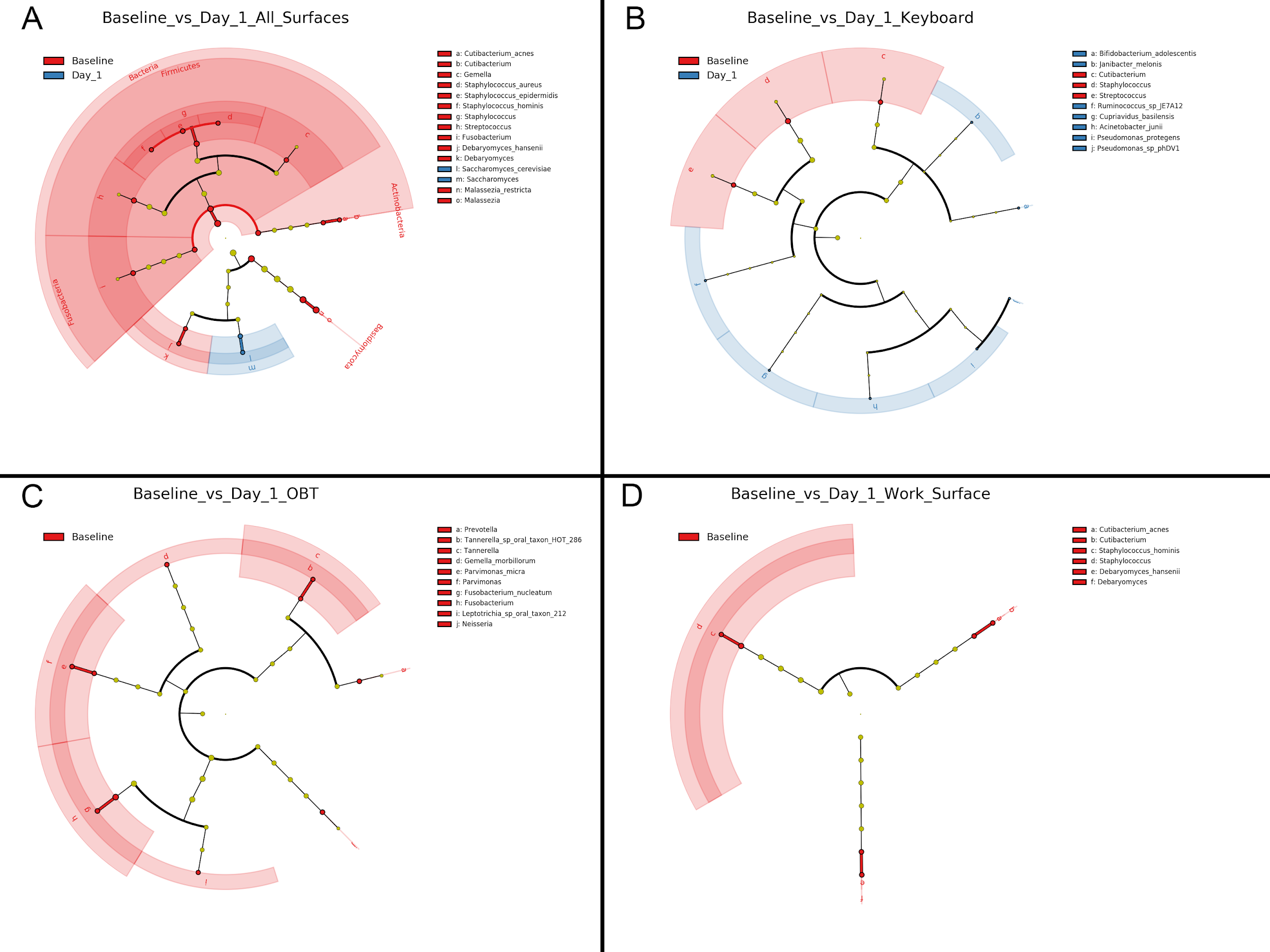


**Supplemental Figure 6.** LEfSe cladograms of differential (Kruskal-Wallis, p≤0.05 and log (LDA)≥1.5) baseline vs. Post taxa (domain, phylum, genus, and species) for the Day 1 Timepoint vs Baseline comparison. (A) All Surfaces, (B) Keyboard, (C) OBT, (D) Work Surfaces. Taxa enriched in Baseline are shown in red, and taxa enriched in Day 1 are shown in blue.


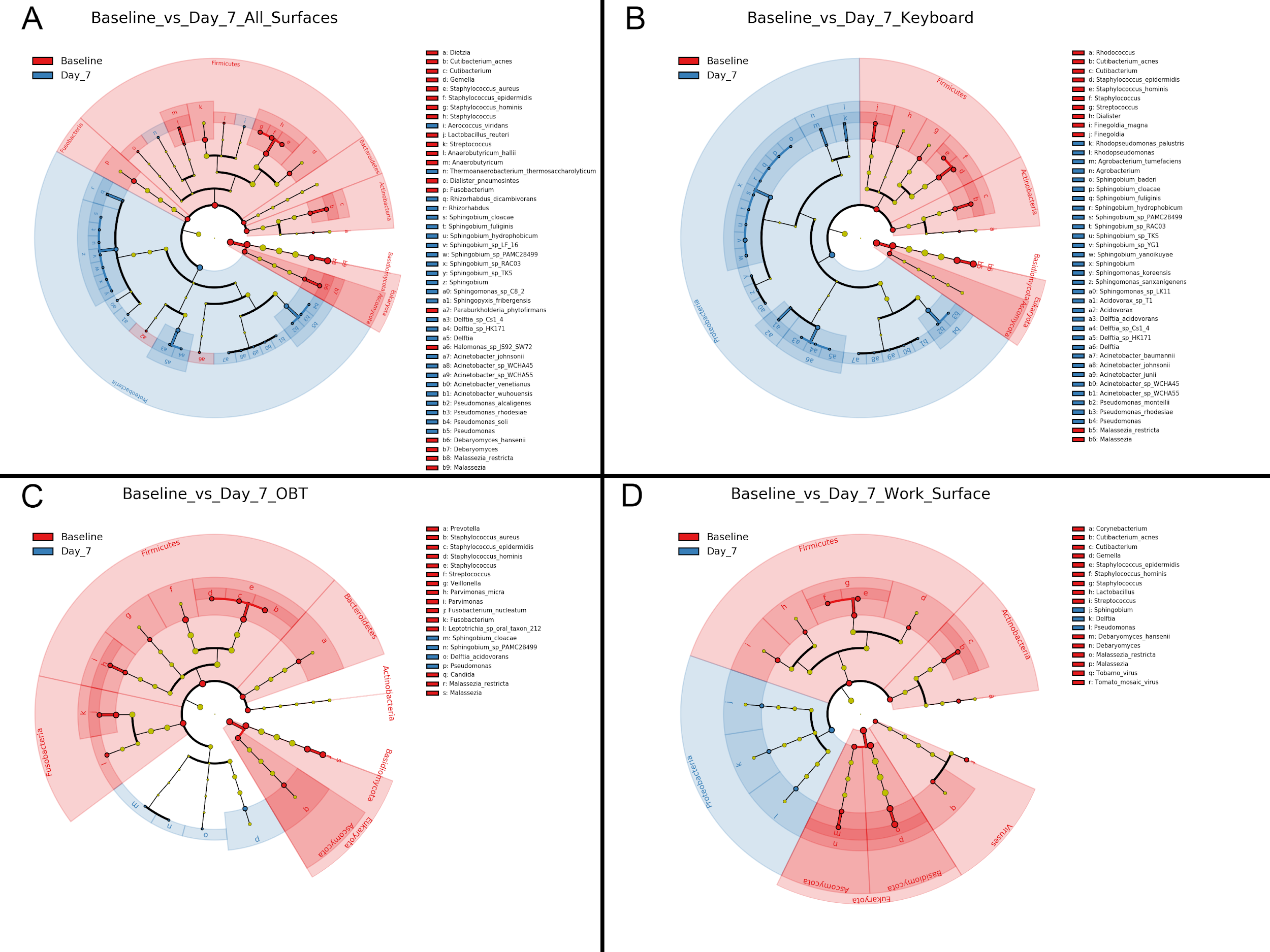


**Supplemental Figure 7.** LEfSe cladograms of differential (Kruskal-Wallis, p≤0.05 and log (LDA)≥1.5) baseline vs. Post taxa (domain, phylum, genus, and species) for the Day 7 Timepoint vs Baseline comparison. (A) All Surfaces, (B) Keyboard, (C) OBT, (D) Work Surfaces. Taxa enriched in Baseline are shown in red, and taxa enriched in Day 7 are shown in blue.


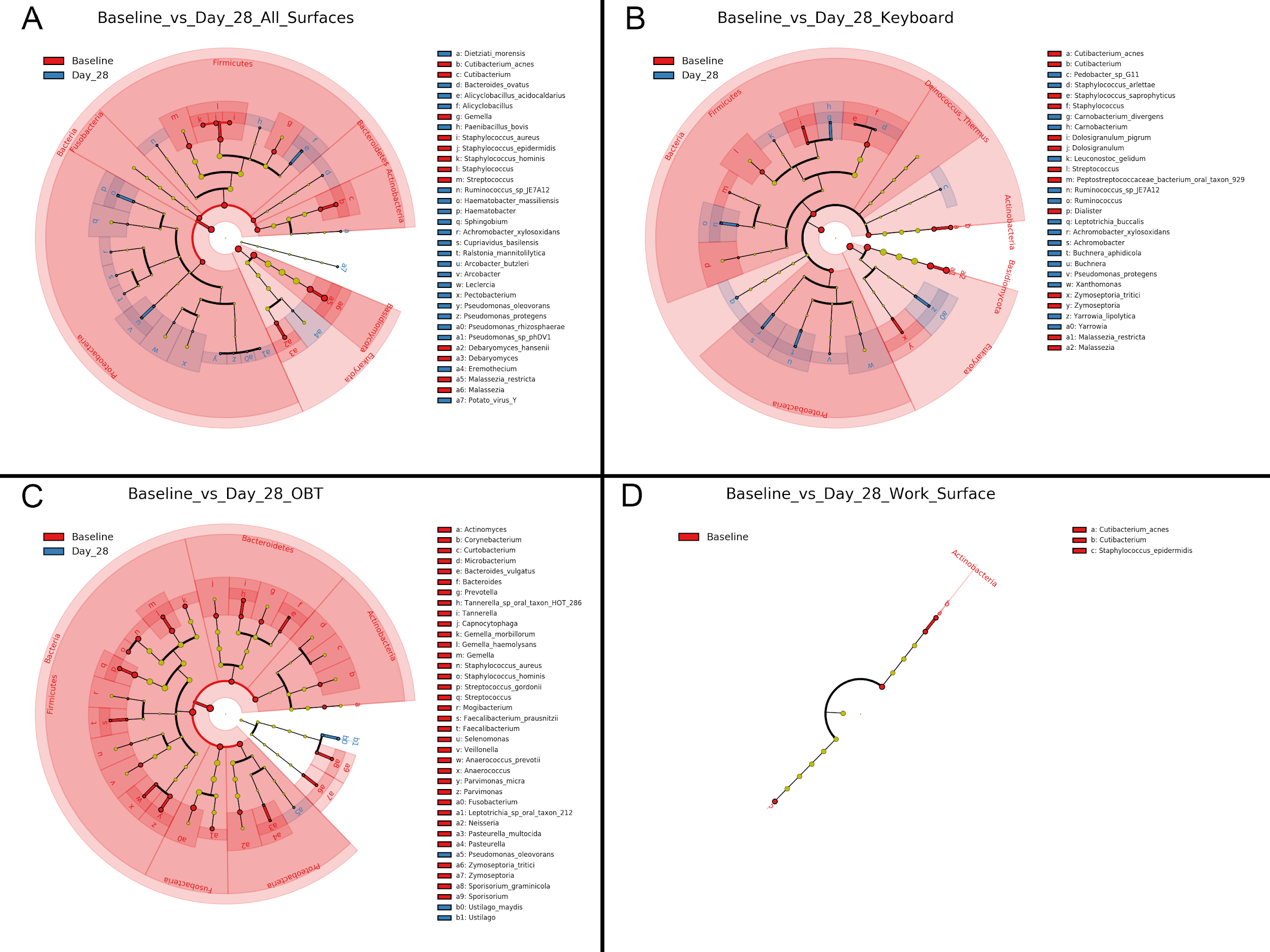


**Supplemental Figure 8.** LEfSe cladograms of differential (Kruskal-Wallis, p≤0.05 and log (LDA)≥1.5) baseline vs. Post taxa (domain, phylum, genus, and species) for the Day 28 Timepoint vs Baseline comparison. (A) All Surfaces, (B) Keyboard, (C) OBT, (D) Work Surfaces. Taxa enriched in Baseline are shown in red, and taxa enriched in Day 28 are shown in blue.


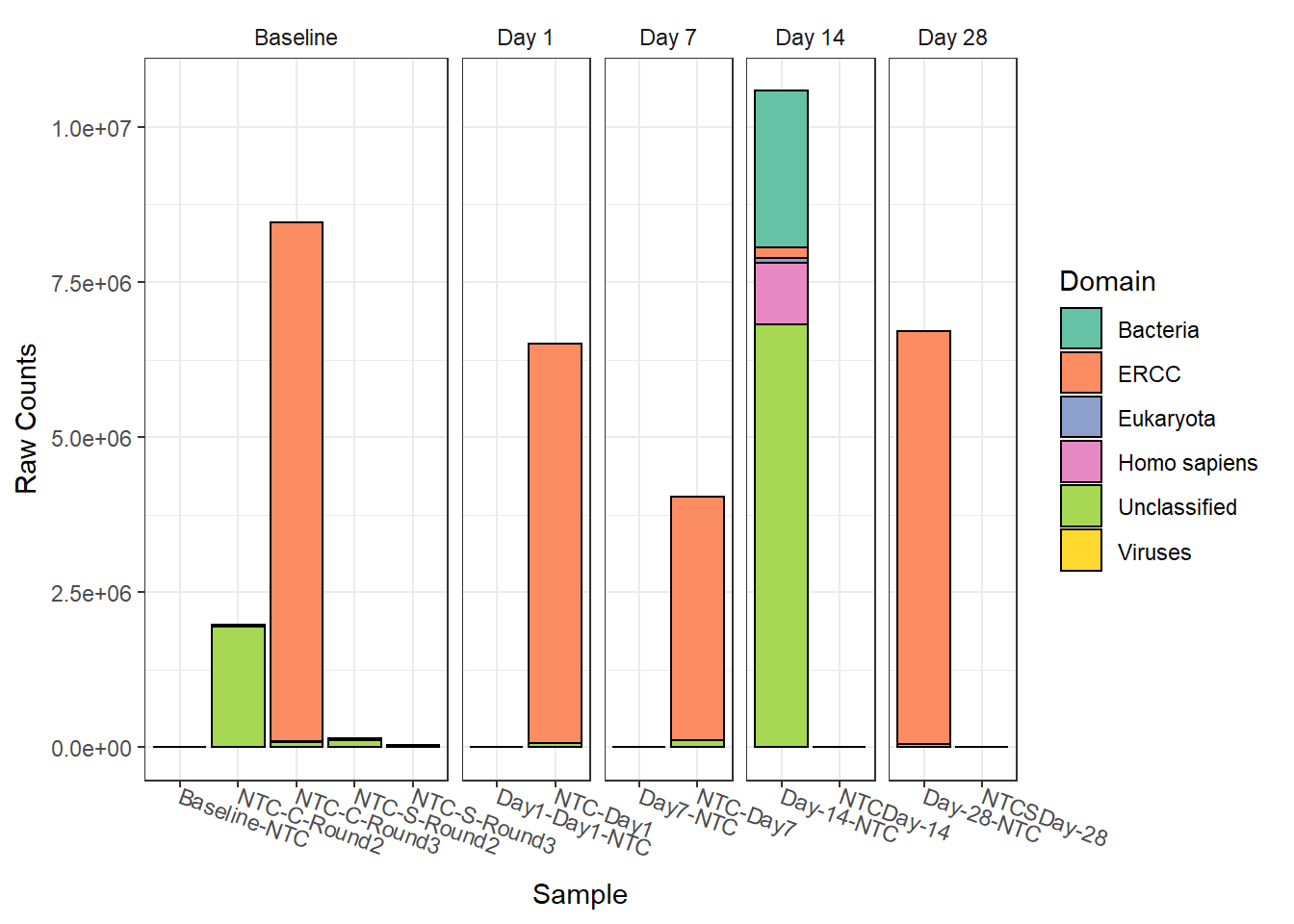


**Supplemental Figure 9.** Barchart of domain-level sequence annotation sequence counts within each non-template control (NTC) sample. Here, we observe the prominent signal within 7/8 NTC samples is internal-control ERCC and unclassified reads. One NTC, processed in union with the Day 14 samples, yielded increased Homo sapiens and microbial annotation counts.


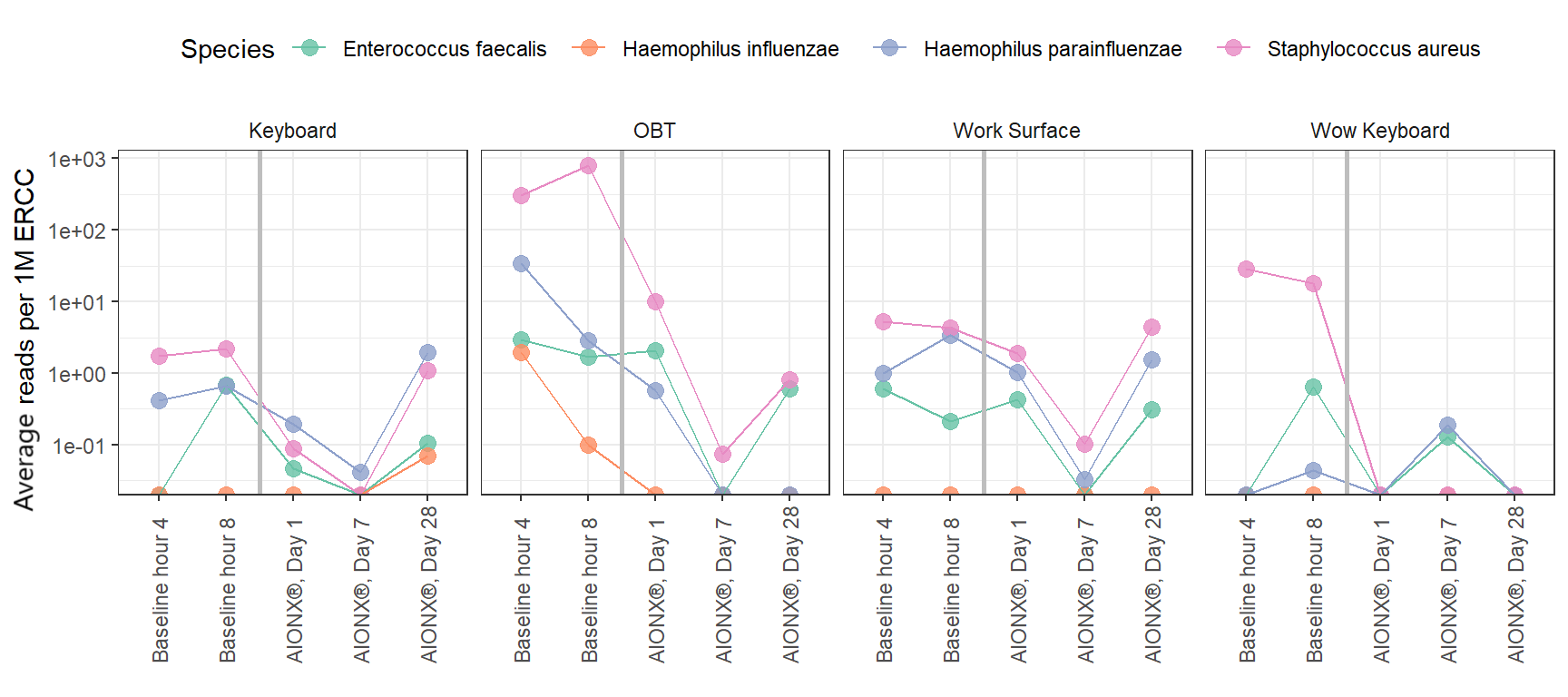


**Supplemental Figure 10.** Normalized average transcript counts of selected biomarker taxa before (Baseline Hour 4, 8) and after the cleanSURFACES® intervention (Day 1, 7, 28) by surface type. Different colored points represent selected biomarker taxa.

**Supplemental Table 1**. RNA and DNA concentrations (ng/uL) and sequence counts for each sample and negative control extracts.

| **Timepoint** | **SampleID** | **RNA (ng/uL)** | **DNA (ng/uL)** | **raw-seqs** | **filtered-seqs** | **rRNA-seqs** | **ERCC-seqs** | **microbial-seqs** |
| --- | --- | --- | --- | --- | --- | --- | --- | --- |
| Baseline (Hour 2) | NTC-Day1 | <0.25 | <0.01 | 7326975 | 6502975 | 380 | 6429405 | 729 |
| Baseline (Hour 4) | DOD00045 | <0.25 | <0.01 | 3961903 | 3076832 | 51820 | 2584207 | 144088 |
| Baseline (Hour 4) | DOD00046 | <0.25 | <0.01 | 3427870 | 2649670 | 178 | 2628356 | 43 |
| Baseline (Hour 4) | DOD00047 | <0.25 | <0.01 | 3675151 | 2868293 | 8409 | 2592005 | 28825 |
| Baseline (Hour 4) | DOD00048 | <0.25 | <0.01 | 3548261 | 2782345 | 1700 | 2725295 | 4615 |
| Baseline (Hour 4) | DOD00049 | <0.25 | <0.01 | 4393706 | 3421093 | 1321 | 3363321 | 2580 |
| Baseline (Hour 4) | DOD00052 | <0.25 | <0.01 | 4086585 | 3229488 | 141 | 3206463 | 41 |
| Baseline (Hour 4) | DOD00053 | <0.25 | <0.01 | 4264900 | 3373334 | 673 | 3343475 | 1232 |
| Baseline (Hour 4) | DOD00054 | <0.25 | <0.01 | 3989262 | 3141062 | 1069 | 3093341 | 4194 |
| Baseline (Hour 4) | DOD00055 | <0.25 | <0.01 | 4074071 | 3129664 | 6326 | 2514319 | 22390 |
| Baseline (Hour 4) | DOD00056 | <0.25 | <0.01 | 3843719 | 3004705 | 3365 | 2890146 | 14382 |
| Baseline (Hour 4) | DOD00057 | <0.25 | <0.01 | 4150704 | 3257531 | 2119 | 3155978 | 8923 |
| Baseline (Hour 4) | DOD00058 | <0.25 | <0.01 | 4033575 | 3153322 | 3905 | 3028946 | 32961 |
| Baseline (Hour 4) | DOD00059 | <0.25 | <0.01 | 3703387 | 2914942 | 1131 | 2859183 | 2146 |
| Baseline (Hour 4) | DOD00060 | <0.25 | <0.01 | 4343197 | 3420142 | 1371 | 3331069 | 3686 |
| Baseline (Hour 4) | DOD00061 | <0.25 | <0.01 | 3879162 | 3044520 | 9603 | 2607763 | 31760 |
| Baseline (Hour 4) | DOD00062 | <0.25 | <0.01 | 4170833 | 3274557 | 2315 | 3188987 | 9252 |
| Baseline (Hour 4) | DOD00063 | <0.25 | <0.01 | 4324141 | 3380104 | 4071 | 3174273 | 14358 |
| Baseline (Hour 4) | DOD00064 | <0.25 | <0.01 | 4210808 | 3312559 | 2866 | 3210816 | 18013 |
| Baseline (Hour 4) | DOD00065 | <0.25 | <0.01 | 4252657 | 3316003 | 14942 | 2729944 | 43745 |
| Baseline (Hour 4) | DOD00066 | <0.25 | <0.01 | 4180833 | 3251271 | 9499 | 2989809 | 62415 |
| Baseline (Hour 8) | DOD00067 | 0.4 | <0.01 | 6836382 | 5308261 | 8755 | 4987967 | 45355 |
| Baseline (Hour 8) | DOD00068 | 0.1 | <0.01 | 7264679 | 5751100 | 908 | 5640167 | 3170 |
| Baseline (Hour 8) | DOD00069 | 0.7 | <0.01 | 7591640 | 6013532 | 22905 | 5052668 | 179571 |
| Baseline (Hour 8) | DOD00070 | 0.2 | <0.01 | 5988614 | 4497576 | 1311 | 4287218 | 4967 |
| Baseline (Hour 8) | DOD00071 | 0.2 | <0.01 | 5774798 | 4568170 | 12542 | 3943510 | 48666 |
| Baseline (Hour 8) | DOD00072 | 0.2 | <0.01 | 6019881 | 4742858 | 811 | 4664689 | 2540 |
| Baseline (Hour 8) | DOD00073 | 0.3 | <0.01 | 8155637 | 6290551 | 11384 | 5723395 | 22287 |
| Baseline (Hour 8) | DOD00074 | 0.2 | <0.01 | 6431675 | 5040820 | 370 | 4947526 | 1143 |
| Baseline (Hour 8) | DOD00075 | 0.3 | <0.01 | 7412083 | 5790532 | 5083 | 5547261 | 11578 |
| Baseline (Hour 8) | DOD00076 | 0.3 | <0.01 | 6030522 | 4704729 | 4319 | 4465709 | 12275 |
| Baseline (Hour 8) | DOD00077 | 0.2 | <0.01 | 6761264 | 5383735 | 3049 | 5244888 | 7894 |
| Baseline (Hour 8) | DOD00078 | 0.2 | <0.01 | 6287302 | 4968836 | 2801 | 4845062 | 14683 |
| Baseline (Hour 8) | DOD00079 | 0.3 | <0.01 | 6294799 | 4882126 | 24320 | 4078907 | 149197 |
| Baseline (Hour 8) | DOD00080 | 0.3 | <0.01 | 6435079 | 5025732 | 2613 | 4905084 | 15904 |
| Baseline (Hour 8) | DOD00081 | 0.1 | <0.01 | 7905983 | 6250831 | 5374 | 5957211 | 27122 |
| Baseline (Hour 8) | DOD00082 | 0.2 | <0.01 | 6155730 | 4845471 | 1784 | 4731306 | 7696 |
| Baseline (Hour 8) | DOD00083 | 0.1 | <0.01 | 6241193 | 4865693 | 3865 | 4618760 | 16683 |
| Baseline (Hour 8) | DOD00084 | 0.2 | <0.01 | 5819972 | 4581371 | 3414 | 4351508 | 26635 |
| Baseline (Hour 8) | DOD00085 | 0.3 | <0.01 | 7095057 | 5568795 | 35494 | 4523051 | 144698 |
| Baseline (Hour 8) | DOD00086 | 0.2 | <0.01 | 6378799 | 4961555 | 19555 | 3996607 | 171467 |
| Baseline (Hour 8) | DOD00087 | 0.3 | <0.01 | 5977853 | 4731510 | 4553 | 4540249 | 24333 |
| Baseline (Hour 8) | DOD00088 | 0.1 | <0.01 | 6160323 | 4849161 | 1124 | 4675676 | 10614 |
| Post (Day 1) | DOD00090 | <0.25 | <0.01 | 7516490 | 6679034 | 9209 | 5495427 | 8026 |
| Post (Day 1) | DOD00091 | <0.25 | <0.01 | 7159489 | 6344521 | 32499 | 5252929 | 166401 |
| Post (Day 1) | DOD00092 | <0.25 | <0.01 | 7394334 | 6613271 | 1429 | 6512969 | 6051 |
| Post (Day 1) | DOD00093 | <0.25 | <0.01 | 7437806 | 6609609 | 17569 | 4681391 | 22600 |
| Post (Day 1) | DOD00094 | <0.25 | <0.01 | 7698576 | 6872994 | 754 | 6768591 | 1308 |
| Post (Day 1) | DOD00095 | <0.25 | <0.01 | 7436352 | 6634467 | 709 | 6533641 | 1093 |
| Post (Day 1) | DOD00096 | <0.25 | <0.01 | 7240744 | 6429543 | 445 | 6359738 | 2173 |
| Post (Day 1) | DOD00097 | <0.25 | <0.01 | 6766449 | 6022164 | 3323 | 5739660 | 20101 |
| Post (Day 1) | DOD00098 | <0.25 | <0.01 | 7365028 | 6555681 | 1178 | 6462856 | 5027 |
| Post (Day 1) | DOD00099 | <0.25 | <0.01 | 6662711 | 5895965 | 8942 | 5424093 | 19204 |
| Post (Day 1) | DOD00100 | <0.25 | <0.01 | 7355366 | 6557313 | 934 | 6481755 | 3131 |
| Post (Day 1) | DOD00101 | <0.25 | <0.01 | 7682281 | 6838995 | 11907 | 6309299 | 36872 |
| Post (Day 1) | DOD00102 | <0.25 | <0.01 | 6874815 | 6116707 | 3662 | 5941141 | 26066 |
| Post (Day 1) | DOD00103 | <0.25 | <0.01 | 7761582 | 6917783 | 1132 | 6786601 | 2872 |
| Post (Day 1) | DOD00104 | <0.25 | <0.01 | 7877840 | 7062046 | 1113 | 6956880 | 1822 |
| Post (Day 1) | DOD00106 | <0.25 | <0.01 | 7783055 | 6967555 | 3543 | 6787538 | 19322 |
| Post (Day 1) | DOD00107 | <0.25 | <0.01 | 7612584 | 6818665 | 1062 | 6698891 | 3927 |
| Post (Day 1) | DOD00108 | <0.25 | <0.01 | 8910279 | 7758516 | 16810 | 7515561 | 31884 |
| Post (Day 1) | DOD00109 | <0.25 | <0.01 | 7001142 | 6247429 | 3999 | 6016070 | 12790 |
| Post (Day 1) | DOD00110 | <0.25 | <0.01 | 6824131 | 6061590 | 5492 | 5851882 | 59304 |
| Post (Day 7) | DOD00112 | <0.25 | <0.01 | 5996027 | 5276574 | 5384 | 5148036 | 8507 |
| Post (Day 7) | DOD00113 | <0.25 | <0.01 | 6796345 | 6044226 | 14339 | 5769974 | 23720 |
| Post (Day 7) | DOD00114 | <0.25 | <0.01 | 6924277 | 6143435 | 7230 | 6002922 | 11929 |
| Post (Day 7) | DOD00115 | <0.25 | <0.01 | 5411919 | 4666500 | 5555 | 4524206 | 8817 |
| Post (Day 7) | DOD00116 | <0.25 | <0.01 | 6249130 | 5415245 | 7546 | 5244177 | 12530 |
| Post (Day 7) | DOD00117 | <0.25 | <0.01 | 5766935 | 5093587 | 4059 | 4937524 | 7174 |
| Post (Day 7) | DOD00118 | <0.25 | <0.01 | 6184509 | 5490331 | 6280 | 5369501 | 10563 |
| Post (Day 7) | DOD00119 | <0.25 | <0.01 | 9303059 | 8249330 | 9752 | 7689480 | 16076 |
| Post (Day 7) | DOD00120 | <0.25 | <0.01 | 5155563 | 4525776 | 2113 | 4421117 | 3695 |
| Post (Day 7) | DOD00121 | <0.25 | <0.01 | 5836561 | 5164318 | 3775 | 5015494 | 7116 |
| Post (Day 7) | DOD00122 | <0.25 | <0.01 | 6838670 | 5991131 | 8879 | 5801754 | 16984 |
| Post (Day 7) | DOD00123 | <0.25 | <0.01 | 6022248 | 5348289 | 3687 | 5225568 | 6576 |
| Post (Day 7) | DOD00124 | <0.25 | <0.01 | 7418322 | 6592463 | 4342 | 6452527 | 9811 |
| Post (Day 7) | DOD00125 | <0.25 | <0.01 | 5075018 | 4456628 | 5313 | 4312496 | 10428 |
| Post (Day 7) | DOD00126 | <0.25 | <0.01 | 11855502 | 10488911 | 10197 | 10223501 | 21859 |
| Post (Day 7) | DOD00127 | <0.25 | <0.01 | 6378095 | 5444221 | 3887 | 5309017 | 7077 |
| Post (Day 7) | DOD00128 | <0.25 | <0.01 | 6024590 | 5300373 | 3275 | 5182698 | 6711 |
| Post (Day 7) | DOD00129 | <0.25 | <0.01 | 6969664 | 6228511 | 4614 | 6129101 | 7161 |
| Post (Day 7) | DOD00130 | <0.25 | <0.01 | 5465320 | 4841393 | 2614 | 4749169 | 5523 |
| Post (Day 7) | DOD00131 | <0.25 | <0.01 | 6469215 | 5669518 | 3927 | 5545233 | 7903 |
| Post (Day 7) | DOD00132 | <0.25 | <0.01 | 5800376 | 5067802 | 1892 | 4975184 | 4835 |
| Post (Day 7) | NTC-Round3 | <0.25 | <0.01 | 11099282 | 8456726 | 408 | 8356825 | 680 |
| Post (Day 7) | NTC-Day7 | <0.25 | <0.01 | 4614878 | 4039256 | 990 | 3915737 | 2720 |
| Post (Day 28) | Day-28-NTC | <0.25 | <0.01 | 8049044 | 6704807 | 485 | 6646906 | 665 |
| Post (Day 28) | DOD00155 | <0.25 | <0.01 | 7838881 | 6507312 | 948 | 6430570 | 1967 |
| Post (Day 28) | DOD00156 | <0.25 | <0.01 | 6784125 | 5605412 | 256 | 5548159 | 215 |
| Post (Day 28) | DOD00157 | <0.25 | <0.01 | 7968903 | 6645472 | 937 | 6446384 | 6127 |
| Post (Day 28) | DOD00158 | <0.25 | <0.01 | 8262219 | 6869437 | 368 | 6767669 | 1501 |
| Post (Day 28) | DOD00159 | <0.25 | <0.01 | 8565618 | 7017156 | 1109 | 6894728 | 3237 |
| Post (Day 28) | DOD00160 | <0.25 | <0.01 | 7446123 | 6154527 | 415 | 6094118 | 1863 |
| Post (Day 28) | DOD00161 | <0.25 | <0.01 | 7728271 | 6434708 | 1511 | 6146247 | 14900 |
| Post (Day 28) | DOD00162 | <0.25 | <0.01 | 7343415 | 6089712 | 274 | 6033267 | 477 |
| Post (Day 28) | DOD00163 | <0.25 | <0.01 | 7500443 | 6206861 | 1525 | 6085019 | 4957 |
| Post (Day 28) | DOD00164 | <0.25 | <0.01 | 6453883 | 5362413 | 831 | 5300398 | 1619 |
| Post (Day 28) | DOD00165 | <0.25 | <0.01 | 6593556 | 5442013 | 3556 | 5198768 | 8928 |
| Post (Day 28) | DOD00166 | <0.25 | <0.01 | 7197824 | 5932136 | 1908 | 5813260 | 9310 |
| Post (Day 28) | DOD00167 | <0.25 | <0.01 | 7767483 | 6446408 | 4688 | 5679499 | 16576 |
| Post (Day 28) | DOD00168 | <0.25 | <0.01 | 7496393 | 6238351 | 2620 | 6071504 | 26922 |
| Post (Day 28) | DOD00169 | <0.25 | <0.01 | 7656025 | 6286576 | 4107 | 5664918 | 15516 |
| Post (Day 28) | DOD00170 | <0.25 | <0.01 | 6785819 | 5588312 | 827 | 5488134 | 8641 |
| Post (Day 28) | DOD00171 | <0.25 | <0.01 | 7978800 | 6515309 | 3900 | 6241463 | 16500 |
| Post (Day 28) | DOD00172 | <0.25 | <0.01 | 6555105 | 5417898 | 1155 | 5237484 | 9759 |
| Post (Day 28) | DOD00173 | <0.25 | <0.01 | 6748886 | 5597907 | 1836 | 5343167 | 7963 |
| Post (Day 28) | DOD00174 | <0.25 | <0.01 | 6820815 | 5671000 | 597 | 5607205 | 4370 |
| Post (Day 28) | DOD00175 | <0.25 | <0.01 | 7865848 | 6460137 | 2315 | 6322181 | 14228 |
| Post (Day 28) | DOD00176 | <0.25 | <0.01 | 7638683 | 6321677 | 1699 | 6222231 | 9927 |
| Post (Day 7) | DOD00111 | <0.25 | <0.01 | 6914800 | 6041896 | 9508 | 5881270 | 14191 |
| Post (Day 1) | DOD00089 | <0.25 | <0.01 | 7811118 | 6959590 | 968 | 6849957 | 1884 |

**Supplemental Table 2.** Normalized taxon annotation count table of all samples observed to pass quality control (QC).
